# Impact of Renal Dysfunction on Mid-Term Outcome after Transcatheter Aortic Valve Implantation: A Systematic Review and Meta-Analysis

**DOI:** 10.1371/journal.pone.0119817

**Published:** 2015-03-20

**Authors:** Chi Chen, Zhen-Gang Zhao, Yan-Biao Liao, Yong Peng, Qing-Tao Meng, Hua Chai, Qiao Li, Xiao-Lin Luo, Wei Liu, Chen Zhang, Mao Chen, De-Jia Huang

**Affiliations:** Department of Cardiology, West China Hospital, Sichuan University, Chengdu, PR China; Sapienza University of Rome, ITALY

## Abstract

**Background:**

There is conflicting evidence regarding the impact of preexisting renal dysfunction (RD) on mid-term outcomes after transcatheter aortic valve implantation (TAVI) in patients with symptomatic aortic stenosis (AS).

**Methods and results:**

Forty-seven articles representing 32,131 patients with AS undergoing a TAVI procedure were included in this systematic review and meta-analysis. Pooled analyses were performed with both univariate and multivariate models, using a fixed or random effects method when appropriate. Compared with patients with normal renal function, mid-term mortality was significantly higher in patients with preexisting RD, as defined by the author (univariate hazard ratio [HR]: 1.69; 95% confidence interval [CI]: 1.50–1.90; multivariate HR: 1.47; 95% CI: 1.17–1.84), baseline estimated glomerular filtration rate (eGFR) (univariate HR: 1.65; 95% CI: 1.47–1.86; multivariate HR: 1.46; 95% CI: 1.24–1.71), and serum creatinine (univariate HR: 1.69; 95% CI: 1.48–1.92; multivariate HR: 1.65; 95% CI: 1.36–1.99). Advanced stage of chronic kidney disease (CKD stage 3–5) was strongly related to bleeding (univariate HR in CKD stage 3: 1.30, 95% CI: 1.13–1.49; in CKD stage 4: 1.30, 95% CI: 1.04–1.62), acute kidney injure (AKI) (univariate HR in CKD stage 3: 1.28, 95% CI: 1.03–1.59; in CKD stage 4: 2.27, 95% CI: 1.74–2.96), stroke (univariate HR in CKD stage 4: 3.37, 95% CI: 1.52–7.46), and mid-term mortality (univariate HR in CKD stage 3: 1.57, 95% CI: 1.26–1.95; in CKD stage 4: 2.77, 95% CI: 2.06–3.72; in CKD stage 5: 2.64, 95% CI: 1.91–3.65) compared with CKD stage 1+2. Patients with CKD stage 4 had a higher incidence of AKI (univariate HR: 1.70, 95% CI: 1.34–2.16) and all-cause death (univariate HR: 1.60, 95% CI: 1.28–1.99) compared with those with CKD stage 3. A per unit decrease in serum creatinine was also associated with a higher mortality at mid-term follow-up (univariate HR: 1.24, 95% CI: 1.18–1.30; multivariate HR: 1.19, 95% CI: 1.08–1.30).

**Conclusions:**

Preexisting RD was associated with increased mid-term mortality after TAVI. Patients with CKD stage 4 had significantly higher incidences of peri-procedural complications and a poorer prognosis, a finding that should be factored into the clinical decision-making process regarding these patients.

## Introduction

As a rapidly evolving procedure, transcatheter aortic valve implantation (TAVI) has been shown to be a safe and effective alternative to surgical aortic valve replacement (SAVR) in high-risk or inoperable patients with symptomatic aortic stenosis (AS) [1–3]. These aging patients frequently have a high prevalence of chronic renal dysfunction (RD), which portends a poor prognosis in those who undergo SAVR [2–4]. However, the results from studies evaluating the impact of baseline renal function on outcomes after TAVI are conflicting [5–7]. In many TAVI studies, although higher mid-term mortality were observed in patients with RD, these differences were not found to be significant by multivariate analyses [6, 8–10]. In addition, the relationship between varying degrees of RD and mid-term prognosis has also not been elucidated.

Therefore, we conducted a meta-analysis of published studies to clarify the mid-term prognostic value of preexisting RD in patients undergoing TAVI.

## Materials and Methods

### Search Strategy

The PubMed online database and the Cochrane library were searched for articles published from January 2002 to April 2014. The following search strategy was used: (transcatheter OR percutaneous OR transfemoral OR transapical OR transsubclavian OR transaortic OR transaxillary) AND (aortic valve) AND (implantation OR replacement) AND (risk factor OR risk assessment OR predictor OR kidney disease OR renal insufficiency OR nephropathy OR creatinine OR estimated glomerular filtration rate OR dialysis OR hemodialysis OR hemodialysis). Reference lists of comparable articles were also retrieved to seek potentially relevant citations.

### Study Selection

Two reviewers conducted the initial screening of titles and abstracts; full-length reports of identified studies were retrieved; and decisions were then made regarding eligibility according to pre-specified inclusion and exclusion criteria. Studies were included if they (1) reported the predictive value of the pre-procedure renal function or mortality outcomes in patients with RD compared with normal controls; (2) performed follow-ups for at least 6 months; and (3) were human studies and published in English. Studies were excluded if they were (1) abstracts, letters, editorials, or reviews and (2) duplicate publications. Studies with overlapping populations were handled by selecting the study that reported on the largest sample of patients undergoing TAVI, unless they used different definitions of RD or reported results in different analysis models.

### Data Extraction

Data were extracted from relevant studies using a pre-specified data collection form that included the first author, journal, year of publication, baseline characteristics, definition of RD, valve type, follow-up duration, and number of complications and deaths. Complications were defined according to the Valve Academic Research Consortium criteria [11], including acute kidney injure (AKI), stroke, all-cause bleeding, and major vascular complications. The outcomes from 6 months to 3 years were defined as mid-term outcomes. The incidence of all-cause mid-term mortality was the primary end point. TAVI-related complications were also the end points of interest.

### Definitions of RD

RD was defined as a diagnosis of chronic kidney disease (CKD), chronic renal failure, renal insufficiency, decreased estimated glomerular filtration rate (eGFR), or elevated serum creatinine level at baseline. CKD stages were classified according to baseline eGFR as follows [12]: ≥60 ml/min (normal or mild CKD, stage 1+2), 30–59 ml/min (moderate CKD, stage 3), 15–29 ml/min (severe CKD, stage 4), and <15 ml/min or dialysis (kidney failure, stage 5). Advanced CKD was defined as CKD stage 3–5.

### Statistical Analysis

The hazard ratio (HR) of preexisting RD with regard to mid-term mortality after TAVI was extracted or calculated. The Generic Invers Variance method in the RevMan software, version 5.20 (The Nordic Cochrane Centre, Copenhagen, Denmark) was used for synthesis of the effect estimates. Heterogeneity was assessed by the *Q*-statistic and *I* ^2^ test. The fixed effects model was selected for the analysis without significant heterogeneity (*I* ^2^<50% and a corresponding *P*>0.1); otherwise, the random effects model was used to obtain the combined effect estimates. Statistical significance was set at *P*<0.05 (two-tailed). Sensitivity analyses were performed using the STATA software version 12.1 (StataCorp, College Station, TX) to test the robustness of the results and the influence of potential effect modifiers. Publication bias was assessed by graphical inspection of funnel plots, Begg’s tests, and Egger’s tests. The “Trim and Fill method” was applied if there was any evidence of publication bias [13].

The present meta-analysis was conducted and reported according to the recommendation of the MOOSE group [14].

## Results

### Study Selection

**Fig 1:**
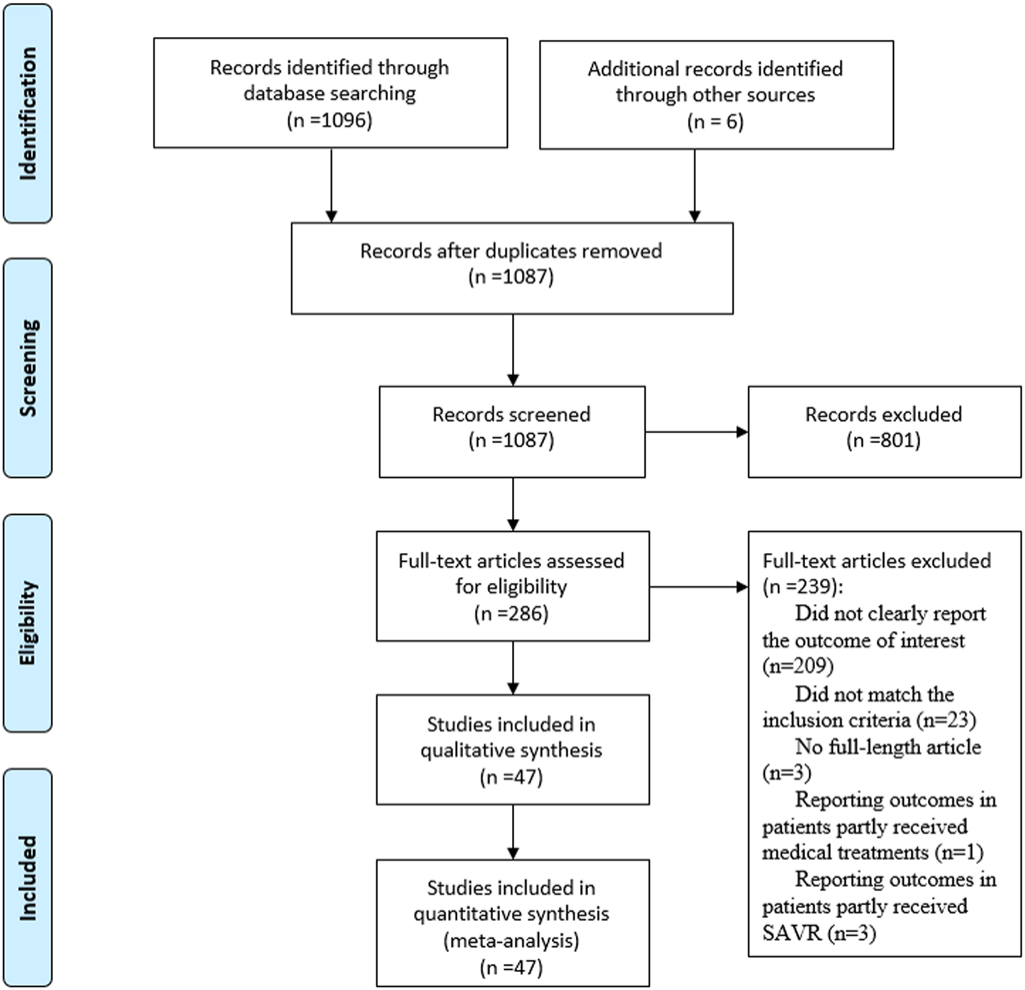
Meta-analysis flow diagram of study selection.

We identified 1096 citations in the initial screening (Fig. 1). After removing duplicates and screening at the abstract level, we retrieved 286 articles for a more detailed evaluation. While 239 studies were subsequently excluded, a total of 47 full-text articles were eligible for this meta-analysis, enrolling a total of 32,131 AS patients with renal function-specific data. No significant limitations were identified for the 47 trials, 3 of which were randomized comparisons [15–17], while others were observational cohort studies [5, 6, 18–56]. Although a few studies had overlapping patient populations, they provided different outcomes according to different definitions of RD, as defined by the author [16, 18, 20, 23, 40, 42, 43], baseline eGFR [10, 27, 49], or serum creatinine [6, 15, 17, 34, 35, 38, 47, 56]. We thus assigned these studies to different groups for either univariate or multivariate analysis.

### Study and Patient Characteristics

The baseline features of the included patients are presented in Table 1. Most studies were conducted in the general population, while a few studies performed TAVI in unique patient groups, such as octogenarians and nonagenarians [43], patients at very high risk (with a EuroSCORE of more than 40%) [42], and patients with chronic lung disease (CLD) [15]. Four studies elucidated the impact of detailed CKD classification on outcomes after TAVI [10, 29, 48, 49]. Patients with end-stage renal disease (ESRD) were included in 14 studies [5, 18, 22–24, 29, 38–41, 43, 47, 53, 55, 56] and were excluded in 7 studies [6, 10, 15–17, 44, 49]. In the remaining studies, the number of patients with ESRD was unclear. Procedural characteristics and the main outcomes after TAVI are summarized in Table 2.

**Table 1:** Baseline characteristics of included patients.

Study	n	Male (%)	Age(y)	STS Score	Logistic Euroscore	Hypertension (%)	Diabetes mellitus (%)	Previous CAD (%)	Previous CE (%)	Renal dysfunction, n (%) / Baseline renal function		LVEF (%)	Mean AVA (cm^2^)	Mean gradient (mmHg)
										Defined	ESRD			
Unbehaun *et al*, 2011 [18]	300	32.3	79.6±8.1	19.1±15.5	38.5±19.4	N/A	24.0	59.3	32.7	Chronic Renal Failure; Baseline eGFR^a^ = 52.0±24.5ml/min/1.73m^2^	Included	EF<25: 9.3	0.7±0.2	48.5±14.9
Tzikas *et al*, 2011 [19]	63	43	82(78–86)	5(3–8)	15(11–19)	57	22	N/A	24	Chronic Renal Disease/Insufficiency; n = 13(21)	Unclear	49±14	N/A	47±19
Sinning *et al*, 2012 [20]	146	47.9	80.5±6.6	9.8±7.3	30.2±18.0	N/A	N/A	61.0	25.3	Chronic Renal Failure; n = 82(56.2)	Unclear	44.5±14.5	0.67±0.15	38.0(29.0–51.0)
Vasa-Nicotera *et al*, 2012 [21]	122	53.3	81.7±6.8	7.3±4.3	22.4±13.0	N/A	N/A	62.3	16.4	Chronic Renal Failure; n = 48(39.3)	Unclear	49.9±10.9	0.64±0.19	44.2±15.8
Wendler *et al*, 2013 [22]	1387	41.5	80.6±7.1	N/A	27.6±16.1	68.9	28.6	55.9	15.2	Renal insufficiency/failure; n = 432(31.2)	18 patients receiving dialysis	EF<30: 5.7	N/A	N/A
Chopard *et al*, 2014 [23]	3933^b^	51	82.8±7.1	14.1±11.7	21.8±14.1	69	26	48	10	Chronic Kidney Disease; n = 336(9)	Included	EF<30: 7.0	N/A	N/A
Muñoz-García *et al*, 2013 [24]	1220^c^	45.3	80.7±6.3	N/A	17.8±13	79.4	31.2	36.1	11.1	Oliguric Renal Failure; n = 17(1.5)	15 patients receiving dialysis	55.8±14	0.62±0.18	51.6±18
Godino *et al*, 2010 [25]	137	53.3	79.5±6.7	7.1±4.6	27.4±16.7	N/A	29.2	N/A	22.6	eGFR<60ml/min/1.73m^2^; n = 51(37.2)	Unclear	50.9±12.6	N/A	52.3±17.3
Rodés-Cabau *et al*, 2010 [5]	339	44.8	81± 8	9.8±6.4	N/A	74.3	23.3	69.0	22.7	eGFR<60ml/min/1.73m^2^; n = 191(56.3); Baseline creatinine = 119±83umol/l	10 patients receiving dialysis	55±14	0.63±0.17	46±17
Hayashida *et al*, 2012 [26]	400	48.5	83.4±6.1	7.9(5.1–12.3)	22.3(17.1–30.3)	69.0	23.0	59.3	10.3	eGFR<60ml/min/1.73m^2^; n = 249(63)	Unclear	54.7±12.3	0.62±0.15	47.8±17.1
Sinning *et al*, 2012 [27]	152	49.3	80.5±6.5	9.8±7.3	30.4±18.1	N/A	N/A	61.2	24.3	eGFR^d^<60ml/min/1.73m^2^; n = 87(57.2); Baseline eGFR = 52±21 ml/min/1.73m^2^	Unclear	44.2±14.5	0.67±0.15	38.0(29.0–51.0)
Nombela-Franco *et al*, 2012 [8]	1061	50.7	81±8	6.5(4.3–9.7)	N/A	74.5	29.4	64.7	18.1	eGFR<60ml/min/1.73m^2^; Baseline eGFR = 60.1±27.8ml/min/1.73m^2^	Unclear	EF<40: 22.1	0.66±0.19	43±16
Kamaga *et al*, 2013 [28]	30	53.3	86±3	N/A	34±12	86.7	20	N/A	16.7	eGFR^d^<60ml/min/1.73m^2^; n = 18(60); Baseline eGFR = 52±17 ml/min/1.73m^2^	Unclear	52±14	N/A	N/A
Dumonteil *et al*, 2013 [29]	942	53.8	81.0±7.0	N/A	20.9(12.9–28.9)	69.5	28.5	45.2	15.7	Chronic Kidney Disease: Mild = 329(eGFR^d^ = 60–89ml/min); Moderate = 399(eGFR = 30–59ml/min); Severe = 72(eGFR <30ml/min)	33 patients receiving dialysis	EF<35: 17	N/A	N/A
Mok *et al*, 2013 [9]	319	46.1	80±8	6.3(4.1–8.9)	N/A	89.0	37.3	63.9	19.1	eGFR<60ml/min/1.73m^2^; n = 192(60.2)	Unclear	54 ± 14	n = 192(60.2)	40±16
Zahn *et al*, 2013 [30]	1318	41.5	81.7±6.1	N/A	20.3±13.5	N/A	34.1	N/A	8.1	eGFR<60ml/min/1.73m^2^; n = 798/1318(60.5)	Unclear	53.5±14.7	0.66±0.24	46. 3±21.8
Urena *et al*, 2014 [31]	1556	47.6	80.2±7.6	7.6±5.3-	20.5±14	81.4	31.2	56.4	N/A	eGFR<60ml/min/1.73m^2^; n = 882 (56.7)	Unclear	55.2±13.9	N/A	47.3±16
Panico *et al*, 2012 [32]	118	46.6	82.5±5.87	N/A	25.8±15.4	80.5	28.8	51.7	5.9	eGFR<60ml/min/1.73m^2^; n = 53(44.9); eGFR<30ml/min/1.73m^2^; n = 9(7.6)	Unclear	EF<30: 5.9	0.75±0.15	50.9±20.6
Katsanos *et al*, 2013 [33]	116	49	81±8	N/A	21.2±12.3	41	27	65	N/A	Creatinine>106umol/l; n = 41(35)	Unclear	54±14	N/A	N/A
Tamburino *et al*, 2011 [34]	663	44	81.0 ± 7.3	N/A	23.0±13.7	75.1	26.4	48.3	7.2	Creatinine>133umol/l; n = 154(23.2)	Unclear	51.2±25.5	N/A	51.8±17.0
Barbanti *et al*, 2014 [35]	518	55.1	81.5±8.4	8.3±5.2	N/A	77.6	30.1	N/A	14.7	Creatinine>177umol/l; n = 197(38)	Unclear	53.9±13.9	0.7±0.4	42.2 ±16.3
Dvir *et al*, 2014 [15]	1108	54.4	82.7±7.2	11.9±4.1	27.2±16.5	N/A	N/A	76.6	28.4	Creatinine>177umol/l; n = 182(16.4)	Excluded	53.1±12.8	0.66±0.19	42.3±14.1
Moat *et al*, 2011 [36]	870	52.4	81.9±7.1	N/A	18.5(11.7–27.9)	N/A	22.8	47.6	N/A	Creatinine>200umol/l; n = 55(6.7)	Unclear	N/A	N/A	N/A
Seiffert *et al*, 2013 [37]	326	44.5	80.6(79.8–81.3)	8.3(7.7–8.9)	22.7(21.2–24.2)	N/A	N/A	61.7	19.3	Creatinine>200umol/l; n = 29(8.9); Baseline creatinine = 1.5(1.3–1.6)mg/dl	Unclear	N/A	0.7(0.7–0.7)	36.5(34.6–38.4)
Luçon et a*l*, 2014 [38]	2435	49.8	83±7	N/A	N/A	69	24.4	47.4	10.4	Creatinine>200umol/l; n = 228(9.4)	63 patients receiving dialysis	52.8±14.6	N/A	47.8±16.8
Heinz *et al*, 2014 [39]	110	46.4	83(58–97)^e^	N/A	10(2–40)^e^	92	N/A	N/A	28	Renal impairment; n = 9(8)	3 patients receiving dialysis	50(11–73) ^e^	N/A	N/A
Web *et al*, 2009 [40]	168	51.8	84(79–87)	9.1(6.3–13.0)	28.6(17.9–41.0)	64.9	23.2	67.9	17.9	Chronic Renal Failure; n = 20(11.9); Baseline creatinine = 98(81–130)umol/l	Included	EF<35: 16.1	0.6(0.5–0.7)	46(34–55)
Ben-Dor *et al*, 2012 [16]	159	42.7	84.4±5.8	11.8±3.9	42.3±21.4	89.3	32.0	57.2	27.0	Chronic Renal Failure; n = 64(40.2); Baseline creatinine = 1.3±1.6 mg/dl	Excluded	51.2±15.9	0.6±0.18	55.3±21.1
Nuis *et al*, 2012 [41]	235	49	80±7	6.1±5.5	19.1±13.7	56	24	N/A	N/A	Chronic Renal Failure; Baseline creatinine = 123±131umol/l	11 patients receiving dialysis	EF<30: 14; 30–59: 35	0.67±0.21	N/A
Drews *et al*, 2013 [42]	186	34.4	81±8	23±14	63±16	N/A	28.5	71.5	42.5	Kidney Failure; Baseline creatinine = 1.5±1.0 mg/dl	Unclear	42±16	0.7±0.2	44±17
Yamamoto *et al*, 2014 [43]	2254	47.5	86.3±3.5	N/A	23.6±16.8	69.9^f^	21.9 ^f^	48.6 ^f^	9.4 ^f^	Renal Insufficiency; n = 200(9.13)^f^	41 patients receiving dialysis	53.7±13.8	0.66±0.18	49.1±16.9
Saia *et al*, 2013 [44]	102	39.2	83.7±5.3	8.2±4.1	22.6±12.4	80.4	22.5	50	4.9	eGFR^a^<30ml/min/1.73m^2^; n = 29(28.4)	Excluded	59.9±11.6	0.6±0.1	46.0±16.7
Conrotto *et al*, 2014 [45]	511	50.5	N/A	N/A	N/A	91.2	29.4	N/A	14.3	eGFR<30ml/min/1.73m^2^; n = 93(18.2)	Unclear	N/A	N/A	N/A
Sinning *et al*, 2010 [6]	77	48	80.8±6.7	9.3±6.1	31.2±17.6	94	23	65	26	eGFR^d^<60ml/min/1.73m^2^; n = 48(62.3); Baseline eGFR = 50.6(38.2±63.8)ml/min/1.73m^2^	Excluded	45.3±16.8	N/A	N/A
Tamburino *et al*, 2012 [46]	218	46.3	80.9±5.2	5.5±4.3	21.1±14.2	85.3	24.3	N/A	13.8	Creatinine>133umol/l; n = 51(23.4)	Unclear	51.1±10.6	0.8±0.2	58.2±16.8
Van Belle *et al*, 2014 [47]	2769	51.1	82.7±7.2	N/A	21.5±13.8	70	25.2	47.1	9.5	Creatinine>200umol/l; n = 233(8.4)	Included	N/A	0.68±0.18	48.4±16.3
Nguyen *et al*, 2013 [48]	321	55.8	82.2±8.2	12.1±7.3	N/A	95.1	43.6	N/A	32.1	Chronic Kidney Disease: Normal/Mild = 159(eGFR^d^≥60ml/min); Moderate = 139(eGFR = 30–59ml/min); Severe = 23(eGFR<30ml/min);	8 patients receiving dialysis	48.2±14.2	N/A	N/A
Yamamoto *et al*, 2013 [49]	642	48.1	83.5±6.5	N/A	22.9±12.2	70.6	22.6	N/A	9.8	Chronic Kidney Disease: Stage1–2 = 218(eGFR^d^≥60ml/min); Stage3a = 182(eGFR = 45–59ml/min); Stage3b = 181(eGFR = 30–44ml/min); Stage4 = 61(eGFR = 15–29ml/min)	Excluded	52.7±14.8	0.64±0.17	47.6±17.5
D'Ascenzm *et al*, 2013 [10]	364	42.3	82.4±5.3	6.6±4.6	23.2±14.1	86.5	31.0	N/A	23.1	Chronic Kidney Disease; Moderate = 219(eGFR^a^ = 30–59ml/min); Severe = 73(eGFR = 15–29ml/min)	Excluded	52.4±11.9	0.62±0.18	53.2±17.3
Lange *et al*, 2012 [50]	420	37	80.3±7.1	6.1±4.1	20.17±13.0	N/A	N/A	55	13.2	Baseline Creatinine = 1.20±0.56 mg/dl	Unclear	EF>50: 62.4; 35–50: 22.1; <35: 15.5	N/A	N/A
Houthuizen *et al*, 2012 [51]	679	47	81(77–85)	N/A	16.0(10.0–25.0)	N/A	23.6	47.0	17.7	Baseline Creatinine = 1.07(0.85–1.38)mg/dl	Unclear	EF<50: 28.0	0.7(0.6–0.8)	4(36–57)
Gotzmann *et al*, 2012 [52]	198	47	80±6	N/A	22±16	N/A	N/A	52	N/A	Baseline Creatinine = 1.2±0.7 mg/dl	Unclear	53±13	0.7±0.1	47±13
Codner *et al*, 2013 [53]	153	37.9	82.1±6.0	9.2±5.3	22.5±13.2	90.2	29.4	N/A	18.3	Baseline eGFR = 66.7±27.3ml/min/1.73m^2^	3 patients with ESRD	N/A	0.62±0.16	50.5±15.4
Sabaté*et al*, 2013 [54]	1416	46	81±6	N/A	17±11	78	34	N/A	10	Baseline Creatinine = 1.26±0.7 mg/dl	Unclear	56±13	0.6±0.2	50±15
Linke *et al*, 2014 [55]	1015	49	81.1± 6.4	5.3(3.6–7.8)	16.0(10.3–25.3)	N/A	31.3	57.8	13.1	Baseline Creatinine = 1.25±0.75 mg/dl; Creatinine clearance<20ml/min = 148(14.9)^g^	Included	53.3±13.7	0.7±0.3	45.6±15.5
Unbehaun *et al*, 2014 [56]	730	39.9	80.1(75.3–84.4)	10.4(6.1–17.8)	28.8 (18.9–48.2)	N/A	29.3	61.2	22.2	Baseline Creatinine Clearance = 53.5(38.9–69.4)ml/min	16 patients receiving dialysis	55.0(40.0–60.0)	0.6(0.6–0.8)	49.5(38.0–57.0)
Kodali *et al*, 2012 [17]	348	57.8	83.6±6.8	11.8±3.3	29.3±16.5	N/A	N/A	74.9	29.3	Baseline Creatinine>2mg/dl; n = 38(11.1)	Excluded	52.5±13.5	0.7±0.2	42.7±14.6

Data are presented as mean±SD or median (interquartile range) as appropriate. Abbreviation used: STS: Society of Thoracic Surgeons; EuroSCORE: European system for cardiac operative risk evaluation; CAD: coronary arterial disease; CE: cerebrovascular event; ESRD: end-stage renal disease; LVEF: left ventricular ejection fraction; AVA: aortic valve area; eGFR: estimated glomerular filtration rate.

a. Calculated by Cockroft-Gault (CG) formula.

b. Mid-term outcomes available in 3597 patients.

c. Data available in 1116 patients.

d. Calculated by Modification of Diet in Renal Disease (MDRD) formula.

e. Data presented as median (minimal to max range).

f. Data available in 2190 patients.

g. Data available in 996 patients.

**Table 2:** Procedure features and main outcomes of included studies.

Study	Approach (%)		Valve type (%)	Follow-up	Peri-procedural complications				Death (%)	Cardiovascular death (%)
	TF	TA			Renal Impairment (%)	Bleeding (%)	MVC (%)	Stroke (%)		
Unbehaun *et al*, 2011 [18]	N/A	N/A	EV: 100	11.7±8.7mo	N/A	1.3	N/A	N/A	65	N/A
Tzikas *et al*, 2011 [19]	N/A	N/A	MCV: 100	383d(356–419)	N/A	N/A	N/A	N/A	28.6	N/A
Sinning *et al*, 2012 [20]	91.8	N/A	MCV: 100	1y	AKI: 23.3	N/A	7.5	5.5	26.7	N/A
Vasa-Nicotera *et al*, 2012 [21]	97.5	1.7	EV: 20.5; MCV: 79.5	1y	N/A	N/A	N/A	N/A	35.2	N/A
Wendler *et al*, 2013 [22]	N/A	100	EV: 100	2y	Dialysis: 6.7	3.9	2.6	2.5	34.9	N/A
Chopard *et al*, 2014 [23]	73	18	EV: 66; MCV: 33	1y	AKI: 1.6	11	9.1	3.3	19.1	8.8
Muñoz-García *et al*, 2013 [24]	91.4	N/A	MCV: 100	238d(50–480)	AKI: 11	N/A	3.9	N/A	10.6	N/A
Godino *et al*, 2010 [25]	78	11	EV: 57.7; MCV: 42.3	6mo	RRT: 8	N/A	16.8	0.7	13.1	5.1
Rodés-Cabau *et al*, 2010 [5]	47.8	52.2	EV: 100	8mo(3–14)	Dialysis: 2.6	N/A	13.3	2.4	22.1	N/A
Hayashida *et al*, 2012 [26]	N/A	N/A	EV: 86.8; MCV: 13.2	279d(101–607)	AKI: 9.0	N/A	8.8	6.5	27.3	N/A
Sinning *et al*, 2012 [27]	92.1	N/A	MCV: 100	1y	AKI: 23.0	9.2	8.6	5.3	27	N/A
Nombela-Franco *et al*, 2012 [8]	68.4	30.3	EV: 64; MCV: 36	12mo(3–23)	N/A	N/A	2.1	N/A	37.8	N/A
Kamaga *et al*, 2013 [28]	100	N/A	EV: 100	1y	AKI: 2.5	N/A	3.3	N/A	26.7	N/A
Dumonteil *et al*, 2013 [29]	84.1	9.3	EV: 46.3; MCV: 53.7	1y	AKI in CKD stage 1+2: 25.7; In CKD stage 3: 23.3; In CKD stage 4: 45.8	CKD stage 1+2: 42.9; CKD stage 3: 56; CKD stage 4: 52.8; CKD stage 5: 42.4	CKD stage 1+2: 6.1; CKD stage 3: 16.7; CKD stage 4: 11; CKD stage 5: 9.6	CKD stage 1+2: 1.8; CKD stage 3: 3; CKD stage 4: 4.2; CKD stage 5: 6.1	18.8	N/A
Mok *et al*, 2013 [9]	39.2	N/A	EV: 98.7	12mo(7–25)	N/A	10.7	N/A	3.1	29.5	14.4
Zahn *et al*, 2013 [30]	88	8.6	EV: 17.9; MCV: 81.5	1y	N/A	N/A	N/A	2.8	21.8	N/A
Urena *et al*, 2014 [31]	N/A	N/A	N/A	22±17mo	N/A	N/A	N/A	N/A	23.4	16.3
Panico *et al*, 2012 [32]	116	N/A	EV: 69.5; MCV: 30.5	1y	AKI: 28.9	22	5.1	7.6	17.8	N/A
Katsanos *et al*, 2013 [33]	41	59	EV: 100	25mo(13–45)	N/A	N/A	N/A	N/A	18.1	N/A
Tamburino *et al*, 2011 [34]	90.3	N/A	MCV: 100	1y	N/A	N/A	1.96	1.2	17.2	N/A
Barbanti *et al*, 2014 [35]	66.2	33.2	EV: 93.2; MCV: 3.1	2y	N/A	N/A	N/A	N/A	22.8	5.8
Dvir *et al*, 2014 [15]	N/A	N/A	N/A	1y	N/A	N/A	N/A	N/A	23.4	10.2
Moat *et al*, 2011 [36]	68.9	N/A	EV: 47.1; MCV: 52.9	1y	N/A	N/A	4	N/A	21.4	N/A
Seiffert *et al*, 2013 [37]	45.7	52.3	EV: 86.2; MCV: 13.8	1y	AKI: 29.4	7.4	8.6	5.8	29.8	18.7
Luçon et a*l*, 2014 [38]	74.9	17.5	EV: 67.3; MCV: 32.7	1y	N/A	N/A	1.6	N/A	16.4	10
Heinz *et al*, 2014 [39]	45	44	N/A	1y	AKI: 55.5	N/A	5	2	27	N/A
Web *et al*, 2009 [40]	79.2	20.7	EV: 100	221d^a^	AKI: 6.0; Dialysis: 1.8	N/A	6.5	4.2	39.1	N/A
Ben-Dor *et al*, 2012 [16]	69.1	30.9	EV: 100	399d(167–669)	N/A	N/A	N/A	N/A	30.8	7.5
Nuis *et al*, 2012 [41]	97	3	MCV: 100	298d(107–688)	AKI: 17	8.9	10.2	4.6	31.1	N/A
Drews *et al*, 2013 [42]	N/A	100	EV: 100)	2y	N/A	N/A	N/A	N/A	46	N/A
Yamamoto *et al*, 2014 [43]	79.06	16.42	EV: 68.5; MCV: 31.5	1y	Dialysis: 1.4	1.2	5	2.5	16.9^b^	N/A
Saia *et al*, 2013 [44]	64.7	23.5	EV: 35.3; MCV: 64.7	1y	AKI: 41.2	4.9	N/A	2	11.8	N/A
Conrotto *et al*, 2014 [45]	57.7	23.3	EV: 53.2; MCV: 46.8	400d(178–715)	AKI: 21.1	43.1	7	1.8	20.4	11.9
Sinning *et al*, 2010 [6]	100	N/A	MCV: 100	1y	AKI: 26	N/A	N/A	N/A	26	N/A
Tamburino *et al*, 2012 [46]	97.2	1.8	EV: 11; MCV: 89	1y	N/A	5.5	N/A	2.3	12.4	N/A
Van Belle *et al*, 2014 [47]	75.3	17.2	EV: 11; MCV: 89	306d(178–490)	N/A	N/A	N/A	N/A	11.3	6.3
Nguyen *et al*, 2013 [48]	62	31	N/A	4 y	Dialysis: 1.9	CKD stage 1+2: 0.6; CKD stage 3: 1.4; CKD stage 4: 0	N/A	CKD stage 1+2: 1.3; CKD stage 3: 1.4; CKD stage 4: 4.4	N/A	N/A
Yamamoto *et al*, 2013 [49]	67.1	N/A	EV: 62.9; MCV: 37.1	1y	AKI in CKD stage 1+2: 13.3; in CKD stage 3: 17.1; in CKD stage 4: 15	N/A	CKD stage 1+2: 7.3; CKD stage 3: 8.3; CKD stage 4: 9.8	CKD stage 1+2: 1.8; CKD stage 3: 3.6; CKD stage 4: 8.2	25.2	N/A
D'Ascenzm *et al*, 2013 [10]	69.5	5.8	N/A	450±250d	AKI in CKD stage 1+2: 8; in CKD stage 3: 14; in CKD stage 4: 18	CKD stage 1+2: 20; CKD stage 3: 22; CKD stage 4: 33	CKD stage 1+2: 10; CKD stage 3: 7; CKD stage 4: 10	CKD stage 1+2: 1.4; CKD stage 3: 2.3; CKD stage 4: 4.1	17.6	10.2
Lange *et al*, 2012 [50]	61	31	EV: 30.6; MCV: 68.7	6mo	N/A	N/A	18.6	4.5	20	N/A
Houthuizen *et al*, 2012 [51]	68.2	30.3	EV: 43; MCV: 57	450d^a^	N/A	N/A	N/A	N/A	28.7	N/A
Gotzmann *et al*, 2012 [52]	N/A	N/A	MCV: 100	535±333d	Dialysis: 2.5	N/A	N/A	2	27.8	16.7
Codner *et al*, 2013 [53]	73.2	17.6	EV: 40.5; MCV: 59.5	2y	AKI: 5.2	2.6	1.3	3.9	11.8	4.6
Sabaté*et al*, 2013 [54]	78.7	21.3	EV: 56.9; MCV: 43.1	244d^c^	AKI^d^: 1.0	2.4	3	2.6	15.9	N/A
Linke *et al*, 2014 [55]	88.4	2.1	MCV: 100	1y	AKI^d^: 6.0	13.8	10.9	3	17.9^e^	11.7^e^
Unbehaun *et al*, 2014 [56]	N/A	100	EV: 100	1.56y(0.40–2.69)	AKI: 18.6; RRT: 3.0	9.7	4	2.3	41.1	N/A
Kodali *et al*, 2012 [17]	244	104	EV: 100	2y	AKI: 1.2	9.3	11	4.7	33.3	19.3

Data are presented as mean±SD or median (interquartile range) as appropriate. Abbreviation used: TF: trans-femoral; TA: trans-apical; MVC: major vascular complications; EV: Edwards Valve; MCV: Medtronic CoreValve; AKI: acute kidney injure; RRT: renal replacement therapy.

a. Data presented as a median.

b. Data available in 2249 patients.

c. Data presented as a mean.

d. Defined as stage 3 according to the Valve Academic Research Consortium (VARC).

e. Data available in 996 patients.

## Mid-Term Outcomes

### Mid-term Mortality according to Different Definitions of RD

#### Defined by the Author

**Fig 2:**
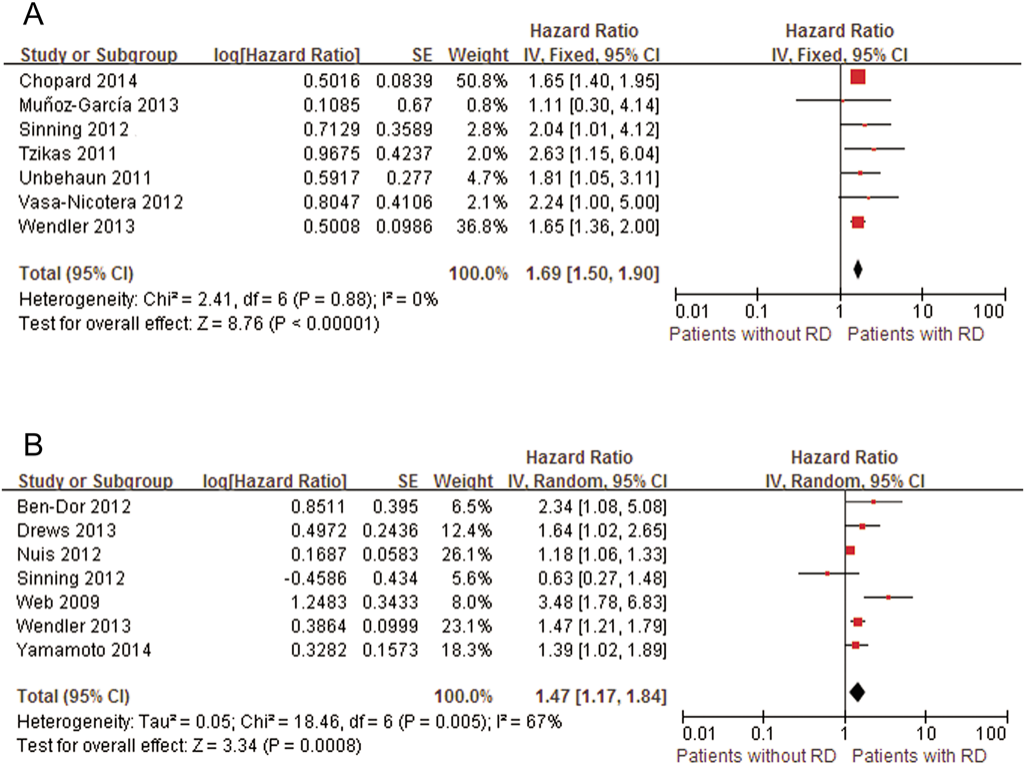
Forest plots of mid-term mortality associated with RD. A, Pooled univariate hazard ratio of patients without RD compared with patients with RD. B, Pooled multivariate hazard ratio of patients without RD compared with patients with RD. RD, renal dysfunction; CI, confidence interval; Fixed, fixed-effects model; Random, Random-effects model; IV, Generic Inverse Variance method.

RD was defined by the author in 12 studies, in which either univariate [18–24] or multivariate [16, 20, 22, 40–43] analysis was performed. These studies enrolled 9769 patients, and the mid-term all-cause mortality rate was 23.6%. Patients with RD had a significantly higher risk for all-cause mortality at the mid-term follow-up (pooled univariate HR: 1.69; 95% CI: 1.50–1.90; pooled multivariate HR: 1.47; 95% CI: 1.17–1.84) compared with patients with normal renal function (Fig. 2). In the univariate model, the results were unchanged when individual studies were omitted or if the study included no more than 100 successful TAVI procedures [19] (pooled univariate HR: 1.67; 95% CI: 1.49–1.88). In the multivariate model, the pooled results also remained stable after removing studies in unique populations, such as patients with a EuroSCORE of more than 40% [42] (pooled multivariate HR: 1.45; 95% CI: 1.13–1.86), octogenarians and nonagenarians [43] (pooled multivariate HR: 1.51; 95% CI: 1.14–1.99), or patients without ESRD [16] (pooled multivariate HR: 1.42; 95% CI: 1.13–1.78).

#### Defined by eGFR

**Fig 3:**
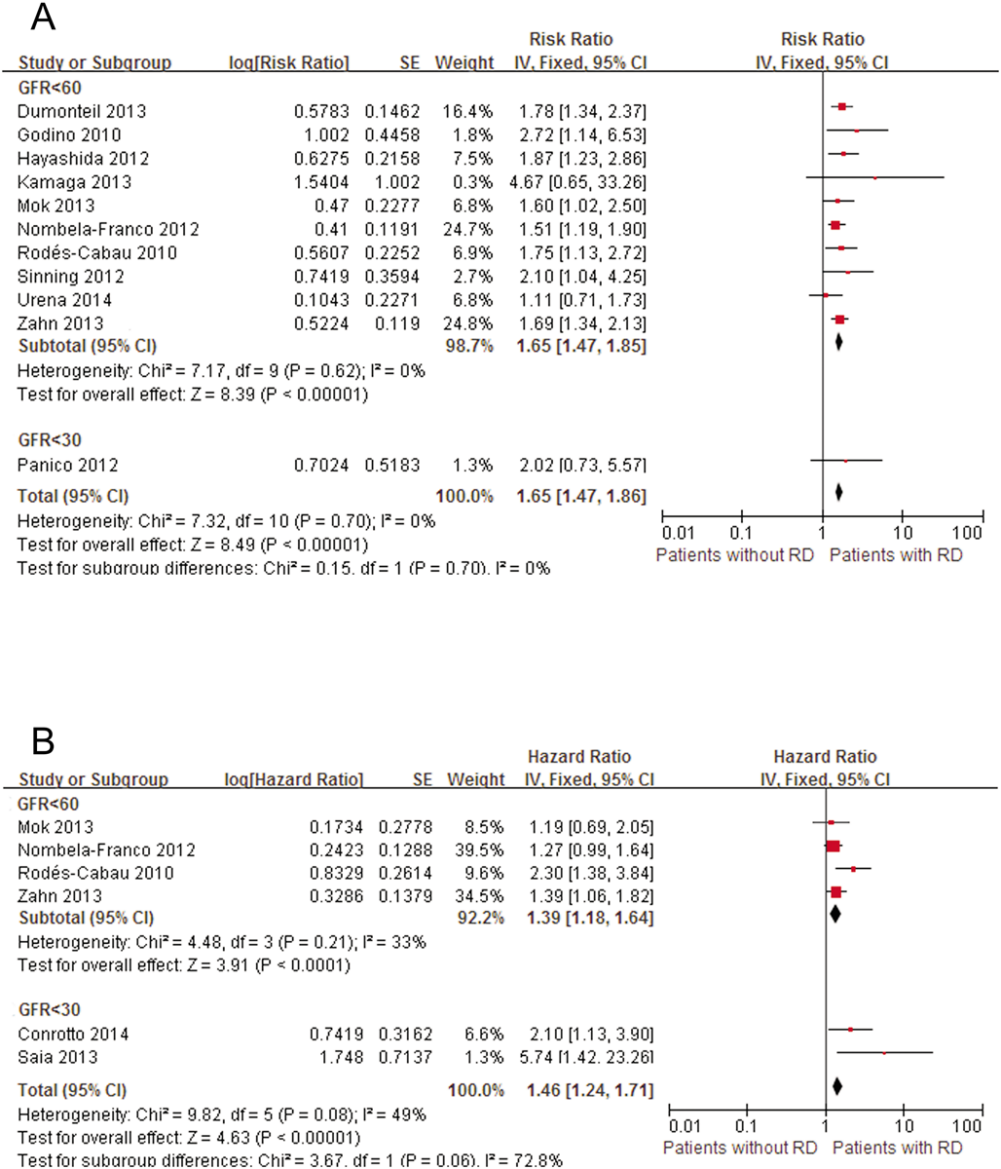
Forest plots of mid-term mortality associated with RD. A, Pooled univariate hazard ratio of patients without RD compared with patients with RD. B, Pooled multivariate hazard ratio of patients without RD compared with patients with RD. RD, renal dysfunction; CI, confidence interval; Fixed, fixed-effects model; IV, Generic Inverse Variance method.

Thirteen studies that included a total of 6,980 patients defined RD as decreased baseline eGFR [5, 8, 9, 25–32, 44, 45]. The mid-term all-cause mortality rate was 24.5%. In patients with RD, mid-term mortality after TAVI was significantly increased compared with that in patients with normal renal function (pooled univariate HR: 1.65; 95% CI: 1.47–1.86; pooled multivariate HR: 1.46; 95% CI: 1.24–1.71) (Fig. 3). In the univariate analysis, the results remained unchanged after excluding the study with a small sample size [28] (pooled univariate HR: 1.65; 95% CI: 1.47–1.85) or the study that focused on patients with a baseline eGFR less than 30 ml/min/1.73 m^2^ [32] (pooled univariate HR: 1.65; 95% CI: 1.47–1.85). Sensitivity analysis of the multivariate model also confirmed the robustness of the results after deleting 2 studies that reported the impact of severe RD (eGFR<30 ml/min/1.73 m^2^) on the mid-term outcomes [44, 45] (pooled multivariate HR: 1.39; 95% CI: 1.18–1.64).

#### Defined by Serum Creatinine

**Fig 4:**
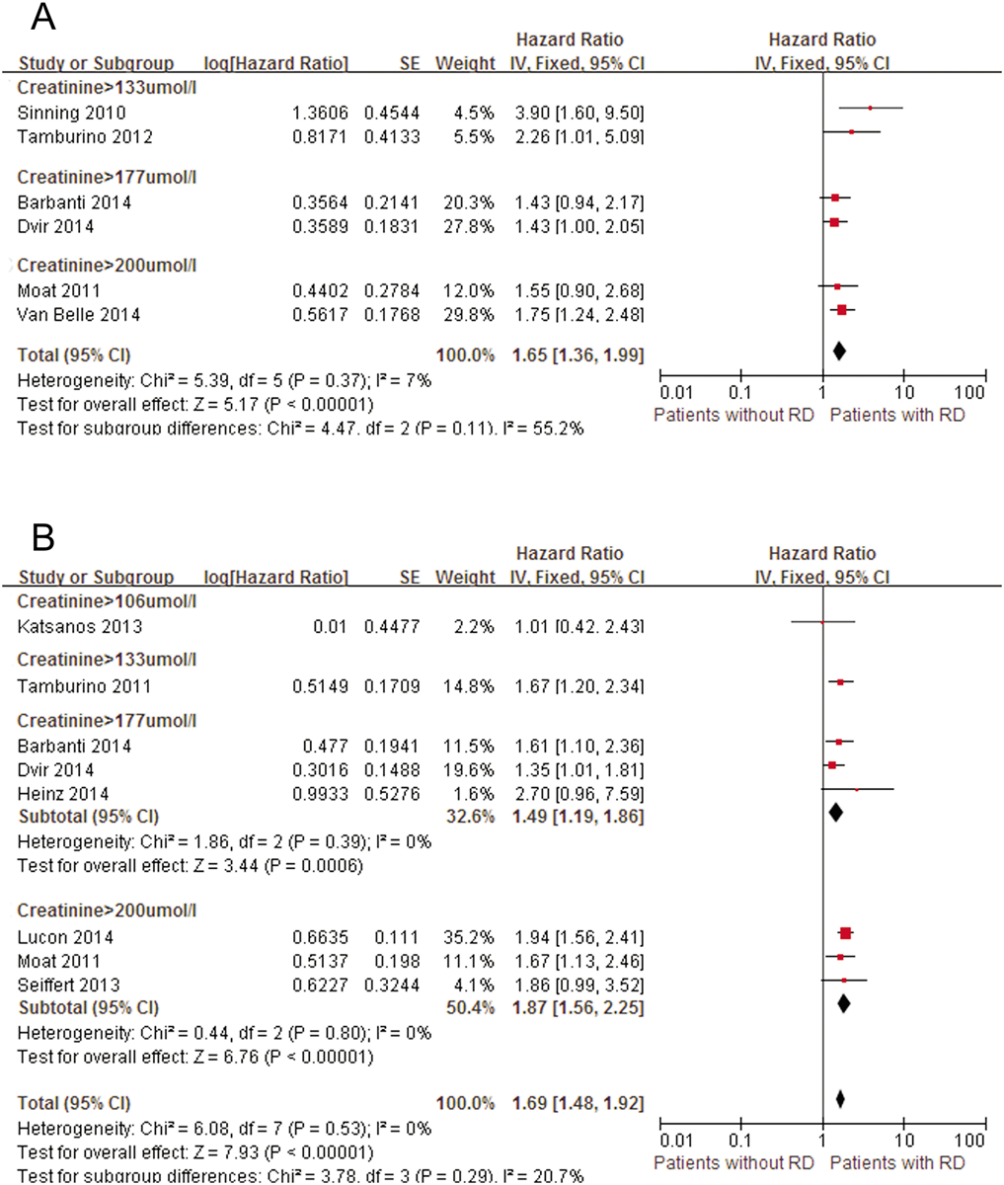
Forest plots of mid-term mortality associated with RD. A, Pooled univariate hazard ratio of patients without RD compared with patients with RD. B, Pooled multivariate hazard ratio of patients without RD compared with patients with RD. RD, renal dysfunction; CI, confidence interval; Fixed, fixed-effects model; IV, Generic Inverse Variance method.

We identified 11 studies with mid-term mortality data in patients with elevated serum creatinine [6, 15, 33–39, 46, 47] (Fig. 4). The cumulative all-cause mortality rate of these 9210 patients was 17.2%. The pooled univariate HR suggested that patients with RD had a significantly higher mid-term mortality rate (pooled univariate HR: 1.69; 95% CI: 1.48–1.92) than patients with normal renal function. These results persisted when omitting individual studies or the study that reported outcomes in the CLD subgroup [15] (pooled univariate HR: 1.78; 95% CI: 1.54–2.05). This relationship was also observed in the multivariate model (pooled multivariate HR: 1.65; 95% CI: 1.36–1.99). After removing the relatively small study [6] (pooled multivariate HR: 1.58; 95% CI: 1.30–1.92) or the study that reported outcomes in the CLD subgroup [15] (pooled multivariate HR: 1.74; 95% CI: 1.39–2.17), the pooled results were still unchanged.

### Association of Mid-term Outcomes with Varying Degrees of RD

Four studies included a detailed classification of CKD according to the baseline eGFR [10, 29, 48, 49], and an additional 4 studies reported the mid-term mortality of patients on chronic dialysis [5, 22, 24, 38].

**Fig 5:**
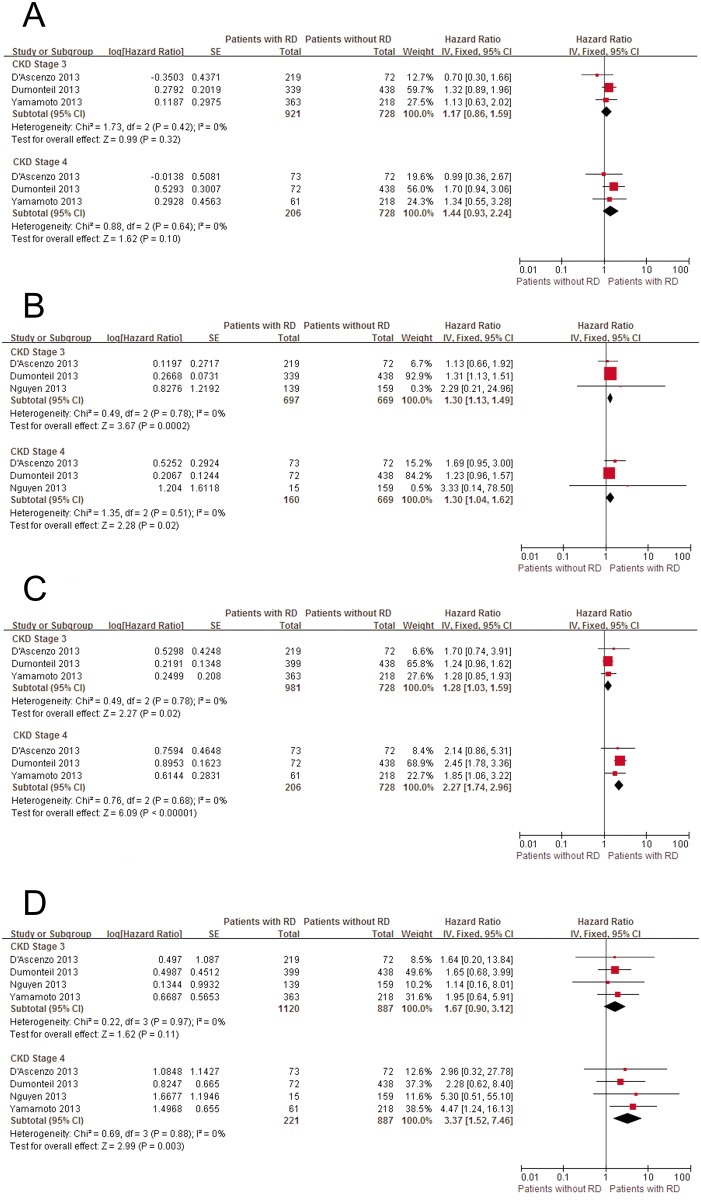
Forest plots of peri-procedural complications associated with RD. A, Pooled univariate hazard ratio of patients without RD compared with patients with RD for all-cause bleeding. B, Pooled univariate hazard ratio of patients without RD compared with patients with RD for major vascular complications. C, Pooled univariate hazard ratio of patients without RD compared with patients with RD for acute kidney injure. D, Pooled univariate hazard ratio of patients without RD compared with patients with RD for stroke. RD, renal dysfunction; CI, confidence interval; Fixed, fixed-effects model; IV, Generic Inverse Variance method.

**Fig 6:**
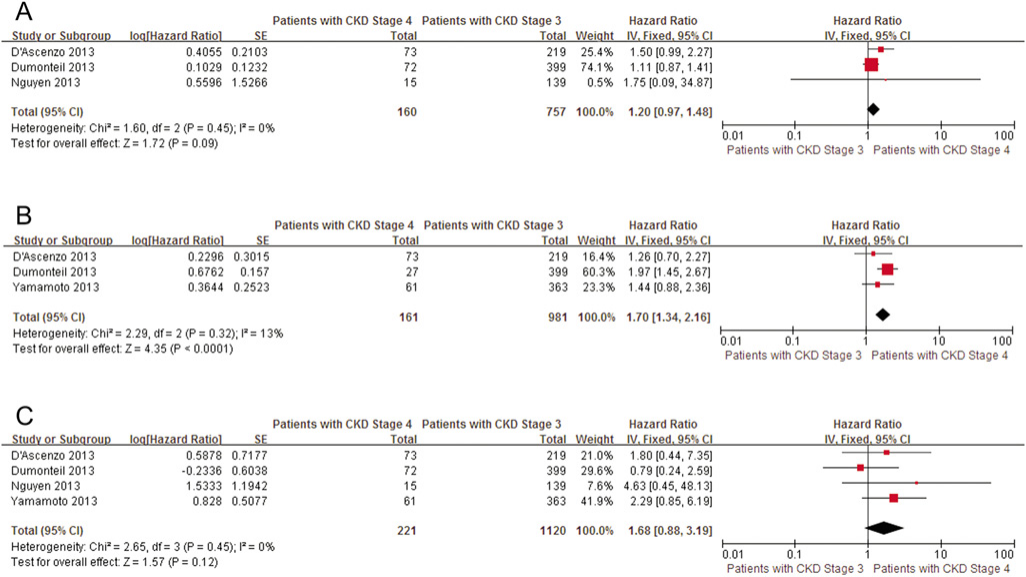
Patients with CKD stage 3 versus patients with CKD stage 4 for peri-procedural complications. A, Pooled univariate hazard ratio of CKD stage 3 compared with CKD stage 4 for all-cause bleeding. B, Pooled univariate hazard ratio of CKD stage 3 compared with CKD stage 4 for acute kidney injure. C, Pooled univariate hazard ratio of CKD stage 3 compared with CKD stage 4 for stroke. RD, renal dysfunction; CI, confidence interval; Fixed, fixed-effects model; IV, Generic Inverse Variance method.

Compared with patients with CKD stage 1+2, patients with advanced CKD had significantly higher incidences of all-cause bleeding (univariate HR in CKD stage 3: 1.30, 95% CI: 1.13–1.49; in CKD stage 4: 1.30, 95% CI: 1.04–1.62), post-procedural AKI (univariate HR in CKD stage 3: 1.28, 95% CI: 1.03–1.59; in CKD stage 4: 2.27, 95% CI: 1.74–2.96), and stroke (univariate HR in CKD stage 4: 3.37, 95% CI: 1.52–7.46). Major vascular complications (MVC) were without significant difference according to baseline renal function status (Fig. 5). Compared with CKD stage 3, CKD stage 4 was strongly related to a higher incidence of AKI ((univariate HR: 1.70, 95% CI: 1.34–2.16), however, this difference was not significant when focusing on bleeding or stroke (Fig. 6). Sensitivity analyses were not conducted due to the small number of studies in each groups.

**Fig 7:**
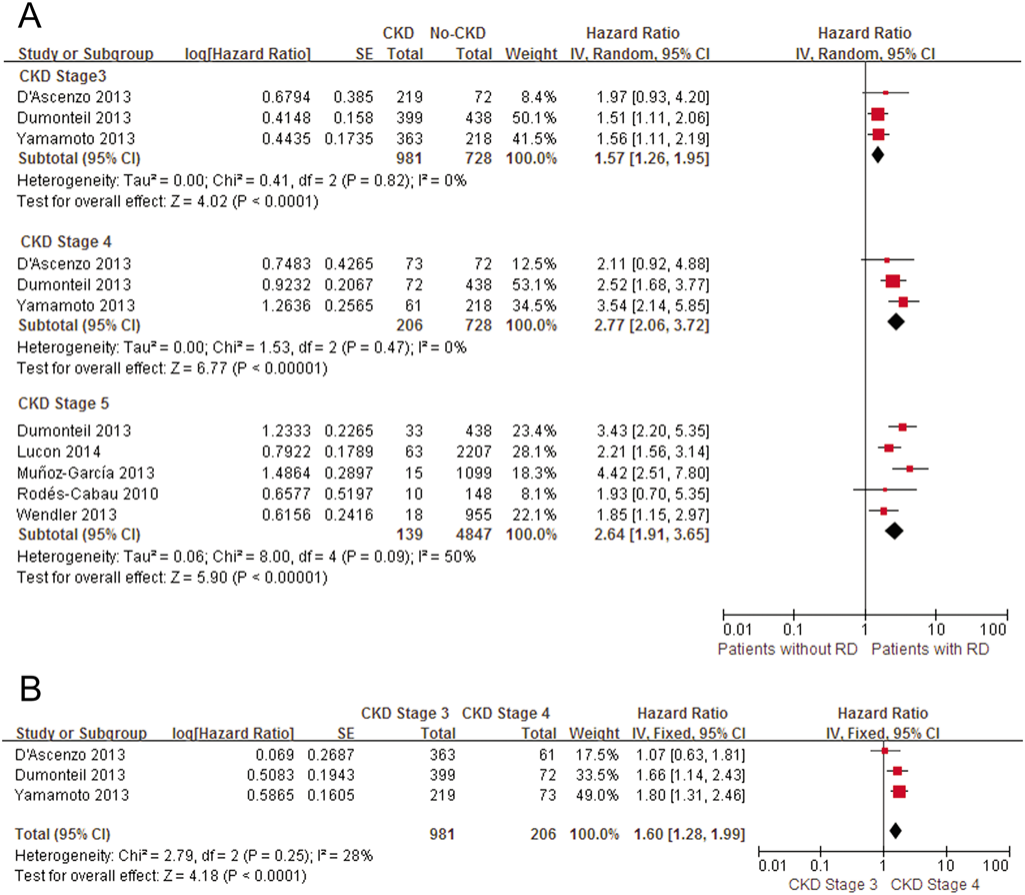
Patients with advanced stages of CKD versus patients with CKD 1+2 for mid-term mortality. A, Pooled univariate hazard ratio of advanced stages of CKD compared with CKD stage 1+2 for all-cause mid-term mortality. B, Pooled multivariate hazard ratio of advanced stages of CKD compared with CKD stage 1+2 for all-cause mid-term mortality. RD, renal dysfunction; CI, confidence interval; Random, Random-effects model; Fixed, fixed-effects model; IV, Generic Inverse Variance method.

At mid-term follow-up, advanced CKD was significantly related to a poorer prognosis compared with CKD stage 1+2 (pooled univariate HR in CKD stage 3: 1.57, 95% CI: 1.26–1.95; in CKD stage 4: 2.77, 95% CI: 2.06–3.72; in CKD stage 5: 2.64, 95% CI: 1.91–3.65). Moreover, compared with patients with CKD stage 3, mortality was significantly increased in patients with CKD stage 4 (pooled univariate HR: 1.60, 95% CI: 1.28–1.99) (Fig. 7). These results persisted after omitting individual studies in the CKD stage 5 group. Due to the small number of studies, sensitivity analyses were not performed in the other groups.

**Fig 8:**
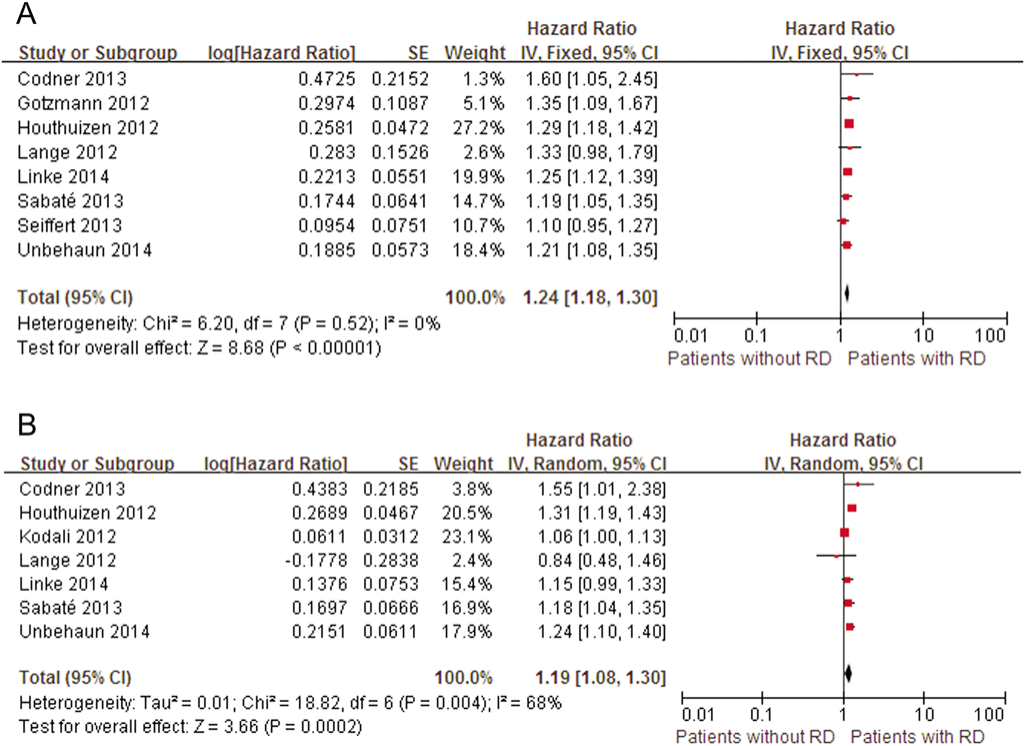
Forest plots of mid-term mortality associated with RD. A, Pooled univariate hazard ratio of patients without RD compared with patients with RD for all-cause mid-term mortality. B, Pooled multivariate hazard ratio of patients without RD compared with patients with RD for all-cause mid-term mortality. RD, renal dysfunction; CI, confidence interval; Fixed, fixed-effects model; Random, Random-effects model; IV, Generic Inverse Variance method.

A total of 9 studies that included 5,266 patients were eligible for the pooled analysis of baseline serum creatinine (for each increase of 1 mg/dl) with respect to mid-term outcomes [18, 37, 50–56] (Fig. 8). The cumulative mortality after TAVI was 24.1%. Each 1 mg/dl increase in serum creatinine significantly raised the mid-term all-cause mortality rate (pooled univariate HR: 1.24, 95% CI: 1.18–1.30; pooled multivariate HR: 1.19, 95% CI: 1.08–1.30). The pooled results remained stable when individual studies in the univariate model were omitted and also persisted in the multivariate analysis after removing the study that excluded patients with ESRD [18] (pooled multivariate HR: 1.24, 95% CI: 1.16–1.32).

The study by Le Ven et al [57] reported a similar finding with regard to baseline eGFR; specifically, each 10 ml/min decrease was found to be associated with a significantly higher risk of all-cause mortality after TAVI (univariate HR: 1.14, 95% CI: 1.07–1.22; multivariate HR: 1.14, 95% CI: 1.07–1.22).

## Publication Bias

Although a subtle publication bias was observed in the funnel plot inspection comparing patients with RD (defined as decreased eGFR) with patients with normal renal function in the univariate model, the pooled estimates remained significant after implementing the “Trim and Fill” method. In the rest of the analyses, funnel plots, Begg’s test and Egger’s test did not provide clear evidence for publication bias (S1–S5 Figs).

## Discussion

The present study is the first to conduct pooled analyses (using both univariate and multivariate models) of the mid-term prognostic value of RD after TAVI. Preexisting RD, despite different definitions, was found to be associated with significantly increased mid-term mortality. Although it has been clearly demonstrated that aging patients with symptomatic AS have a high prevalence of RD, only a few TAVI studies have treated RD as a component of the primary study question, and the results have been conflicting [6, 7, 10, 29, 44, 48, 49, 58]. By conducting this meta-analysis, we have clearly shown a correlation between mid-term outcome and baseline renal function, as reflected by either baseline eGFR or serum creatinine.

Varying degrees of RD, as classified by advanced stages of CKD, were associated with significantly higher incidences of bleeding, AKI, and mid-term mortality after TAVI. Post-procedural stroke occurred more frequently in patients with CKD stage 4 compared with CKD 1+2. These findings were in line with previous TAVI studies focusing on the peri-procedural complications [8, 44, 59]. However, differences about the incidence of MVC were not significant in our study. At mid-term follow-up, patients with CKD stage 4 were noted to have a poorer prognosis compared with patients with CKD stage 3. This graded association was further confirmed when considering baseline serum creatinine (for each 1 mg/mL decrease), which was also strongly related to increased mid-term mortality. In previous studies, advanced stages of CKD have been shown to be independent risk factors in patients undergoing TAVI [10, 29, 49]. However, no stepwise increased adverse events was observed in patients with more severe CKD [10, 29]. By pooling estimate effects from these individual studies, we found that patients with CKD stage 4 had significantly higher incidence of AKI and mortality rates compared with those with CKD stage 3. Because patients with ESRD have been excluded from many TAVI studies, only sparse data exist on the prognostic value of CKD stage 5 [29, 60]. In the present study, pre-procedural chronic dialysis was also shown to be a strong risk factor for mid-term mortality after TAVI.

The presence of RD is an important factor contributing to poorer outcomes in patients undergoing TAVI. This phenomenon can be explained by several aspects. (1) Patients with RD were older, were frailer, and presented with a significantly higher Logistic Euroscore in previous studies [10, 29, 49]. In view of these data, RD may serve as a marker of unbalanced baseline risk profiles. Patients with RD frequently have a higher burden of severe morbidities, which may adversely affect their survival after TAVI. (2) RD modifies the natural history of AS, presumably through a pathophysiological mechanism that promotes calcium deposition on aortic leaflets, thereby worsening aortic stenosis and reducing cardiac output [61]. Severe AS with decreased flow to important organs is responsible for the onset of severe complications, which subsequently increase the mortality after TAVI [8, 48, 62]. RD was also found to be associated with disorders of primary hemostasis, in particular platelet malfunctions [63], which played an important role in the occurrence of peri-procedural bleeding and subsequently increased mortality [59]. (3) It is well known that one of the advantages of TAVI is the avoidance of cardiopulmonary bypass, which is one of the most important risk factors for post-procedural AKI [64]. However, the incidence of contrast-induced nephropathy (CIN) could conceivably increase as a result of the extensive use of contrast medium and multiple injections [65, 66]. Although few studies have identified a significant association between contrast agents and AKI in the general population [67, 68], when focusing on patients with RD, the incidence of CIN was found to be significantly enhanced. Among patients with CKD, the occurrence of CIN was strongly associated with a higher 60-day mortality [69], indicating that the nephrotoxic mechanisms of CIN were one of the major issues contributing to the mid-term mortality in such patients.

In patients with more severe kidney failure, a higher Logistic Euroscore and lower ejection fraction were more frequent [10, 29]. Moreover, the incidence of post-procedural renal impairment was also significantly higher in patients with more severe RD, despite using a smaller dose of contrast medium [29]. These results could explain the graded association between the severity of RD and the stepwise increase in mortality after TAVI.

In view of these data, RD appears to be not only a marker of illness severity, but it also represents a risk factor for mid-term prognosis. Therefore, rigorous risk assessment, preventive therapies for bleeding and stroke, and timely detection of AKI would be crucial interventions that would improve the mid-term mortality after TAVI. Our study revealed higher incidence of peri-procedural complications and poorer outcomes in patients with CKD stage 4. However, this result also raises questions regarding whether these high-risk patients actually benefit from a TAVI procedure and which patients are at the highest risk of mid-term mortality.

## Limitations

Several limitations exist in our study. (1) Because the present meta-analysis was based only on published studies, the possibility of potential publication bias cannot be completely ruled out. (2) Although careful screening was conducted, the possibility of overlapping study populations could result in similar estimates. (3) Our meta-analysis was not conducted at the patient level, and only 5 studies treated RD as the primary study question. Even though the renal function-specific baseline characteristics were not available, the effects of comorbidities could not be assessed. (4) The adjusted prognostic value of different degrees of RD on the mid-term mortality after TAVI was not assessed due to the scarcity of study data. (5) Most included studies calculated eGFR using the MDRD equation, which is affected by the considerable decline in muscle mass with age, severe cardiovascular disease, drugs, and diet, making it difficult to reflect the actual renal clearance in the cohort of elderly patients.

## Conclusions

Preexisting RD, despite different definitions, was associated with significantly increased mid-term mortality after TAVI. Varying degrees of RD were strongly associated with a stepwise increase in mid-term mortality rates. Given that patients with CKD stage 4 had a higher incidence of peri-procedural complications and a poorer prognosis, TAVI in such patients may present a significant challenge.

## Supporting Information

S1 Checklist (DOC)

S1 Fig Funnel plots of comparison between RD (defined by the author) and normal renal function for mid-term mortality. A, Comparison in univariable model (Begg’s test: P = 0.23; Egger’s test: P = 0.208; Trim and Fill Analysis not performed). B, Comparison in multivariable model. (Begg’s test: P = 0.548; Egger’s test: P = 0.215; Trim and Fill Analysis not performed).
(TIF)

S2 Fig Funnel plots of comparison between RD (defined as decreased eGFR) and normal renal function for mid-term mortality. A, Comparison in univariable model. (Begg’s test: P = 0.119; Egger’s test: P = 0.129; Trim and Fill Analysis not performed). B, Comparison in multivariable model. (Begg’s test: P = 0.133; Egger’s test: P = 0.06; Trim and Fill Analysis: Pooled estimate = 0.306, P<0.001).
(TIF)

S3 Fig Funnel plots of comparison between RD (defined as increased Serum creatinine) and normal renal function for mid-term mortality. A, Comparison in univariable model. (Begg’s test: P = 0.711; Egger’s test: P = 0.711; Trim and Fill Analysis not performed). B, Comparison in multivariable model. (Begg’s test: P = 0.133; Egger’s test: P = 0.086; Trim and Fill Analysis: Pooled estimate = 0.436, P<0.001).
(TIF)

S4 Fig Funnel plots of comparison between CKD stage 5 and CKD stage 1+2 for mid-term mortality. Begg’s test: P = 0.806; Egger’s test: P = 0.841; Trim and Fill Analysis not performed.
(TIF)

S5 Fig Funnel plots for the impact of baseline serum creatinine (for each increase of 1 mg/dl) on the mid-term mortality. A, Preformed in univariable model. (Begg’s test: P = 0.902; Egger’s test: P = 0.430; Trim and Fill Analysis not performed). B, Preformed in multivariable model. (Begg’s test: P = 0.764; Egger’s test: P = 0.507; Trim and Fill Analysis not performed).
(TIF)

## References

[1] Smith CR, Leon MB, Mack MJ, Miller DC, Moses JW, Svensson LG, et al Transcatheter versus surgical aortic-valve replacement in high-risk patients. N Engl J Med. 2011;364: 2187–98. 10.1056/NEJMoa1103510 21639811

[2] Leon MB, Smith CR, Mack M, Miller DC, Moses JW, Svensson LG, et al Transcatheter aortic-valve implantation for aortic stenosis in patients who cannot undergo surgery. N Engl J Med. 2010;363: 1597–607. 10.1056/NEJMoa1008232 20961243

[3] Makkar RR, Fontana GP, Jilaihawi H, Kapadia S, Pichard AD, Douglas PS, et al Transcatheter aortic-valve replacement for inoperable severe aortic stenosis. N Engl J Med. 2012;366: 1696–704. 10.1056/NEJMoa1202277 22443478

[4] Thourani VH, Keeling WB, Sarin EL, Guyton RA, Kilgo PD, Dara AB, et al Impact of preoperative renal dysfunction on long-term survival for patients undergoing aortic valve replacement. Ann Thorac Surg. 2011;91: 1798–806; discussion 806–7. 10.1016/j.athoracsur.2011.02.015 21536247

[5] Rodes-Cabau J, Webb JG, Cheung A, Ye J, Dumont E, Feindel CM, et al Transcatheter aortic valve implantation for the treatment of severe symptomatic aortic stenosis in patients at very high or prohibitive surgical risk: acute and late outcomes of the multicenter Canadian experience. J Am Coll Cardiol. 2010;55: 1080–90. 10.1016/j.jacc.2009.12.014 20096533

[6] Sinning JM, Ghanem A, Steinhauser H, Adenauer V, Hammerstingl C, Nickenig G, et al Renal function as predictor of mortality in patients after percutaneous transcatheter aortic valve implantation. JACC Cardiovasc Interv. 2010;3: 1141–9. 10.1016/j.jcin.2010.09.009 21087750

[7] Wessely M, Rau S, Lange P, Kehl K, Renz V, Schonermarck U, et al Chronic kidney disease is not associated with a higher risk for mortality or acute kidney injury in transcatheter aortic valve implantation. Nephrol Dial Transplant. 2012;27: 3502–8. 10.1093/ndt/gfs102 22535634

[8] Nombela-Franco L, Webb JG, de Jaegere PP, Toggweiler S, Nuis RJ, Dager AE, et al Timing, predictive factors, and prognostic value of cerebrovascular events in a large cohort of patients undergoing transcatheter aortic valve implantation. Circulation. 2012;126: 3041–53. 10.1161/CIRCULATIONAHA.112.110981 23149669

[9] Mok M, Nombela-Franco L, Dumont E, Urena M, DeLarochelliere R, Doyle D, et al Chronic obstructive pulmonary disease in patients undergoing transcatheter aortic valve implantation: insights on clinical outcomes, prognostic markers, and functional status changes. JACC Cardiovasc Interv. 2013;6: 1072–84. 10.1016/j.jcin.2013.06.008 24156967

[10] D'Ascenzo F, Moretti C, Salizzoni S, Bollati M, D'Amico M, Ballocca F, et al 30 days and midterm outcomes of patients undergoing percutaneous replacement of aortic valve according to their renal function: a multicenter study. Int J Cardiol. 2013;167: 1514–8. 10.1016/j.ijcard.2012.04.161 22726400

[11] Leon MB, Piazza N, Nikolsky E, Blackstone EH, Cutlip DE, Kappetein AP, et al Standardized endpoint definitions for transcatheter aortic valve implantation clinical trials: a consensus report from the Valve Academic Research Consortium. Eur Heart J. 2011;32: 205–17. 10.1093/eurheartj/ehq406 21216739 PMC3021388

[12] Levey AS, Eckardt KU, Tsukamoto Y, Levin A, Coresh J, Rossert J, et al Definition and classification of chronic kidney disease: a position statement from Kidney Disease: Improving Global Outcomes (KDIGO). Kidney Int. 2005;67: 2089–100. 15882252 10.1111/j.1523-1755.2005.00365.x

[13] Duval S, Tweedie R. Trim and fill: A simple funnel-plot-based method of testing and adjusting for publication bias in meta-analysis. Biometrics. 2000;56: 455–63. 10877304 10.1111/j.0006-341x.2000.00455.x

[14] Stroup DF, Berlin JA, Morton SC, Olkin I, Williamson GD, Rennie D, et al Meta-analysis of observational studies in epidemiology: a proposal for reporting. Meta-analysis Of Observational Studies in Epidemiology (MOOSE) group. Jama. 2000;283: 2008–12. 10789670 10.1001/jama.283.15.2008

[15] Dvir D, Waksman R, Barbash IM, Kodali SK, Svensson LG, Tuzcu EM, et al Outcomes of patients with chronic lung disease and severe aortic stenosis treated with transcatheter versus surgical aortic valve replacement or standard therapy: insights from the PARTNER trial (placement of AoRTic TraNscathetER Valve). J Am Coll Cardiol. 2014;63: 269–79. 10.1016/j.jacc.2013.09.024 24140659

[16] Ben-Dor I, Dvir D, Barbash IM, Okubagzi P, Torguson R, Xue Z, et al Outcomes of patients with severe aortic stenosis at high surgical risk evaluated in a trial of transcatheter aortic valve implantation. Am J Cardiol. 2012;110: 1008–14. 10.1016/j.amjcard.2012.05.034 22721576

[17] Kodali SK, Williams MR, Smith CR, Svensson LG, Webb JG, Makkar RR, et al Two-year outcomes after transcatheter or surgical aortic-valve replacement. N Engl J Med. 2012;366: 1686–95. 10.1056/NEJMoa1200384 22443479

[18] Unbehaun A, Pasic M, Drews T, Dreysse S, Kukucka M, Hetzer R, et al Analysis of survival in 300 high-risk patients up to 2.5 years after transapical aortic valve implantation. Ann Thorac Surg. 2011;92: 1315–23. 10.1016/j.athoracsur.2011.05.077 21958779

[19] Tzikas A, Geleijnse ML, Van Mieghem NM, Schultz CJ, Nuis RJ, van Dalen BM, et al Left ventricular mass regression one year after transcatheter aortic valve implantation. Ann Thorac Surg. 2011;91: 685–91. 10.1016/j.athoracsur.2010.09.037 21352980

[20] Sinning JM, Hammerstingl C, Vasa-Nicotera M, Adenauer V, Lema Cachiguango SJ, Scheer AC, et al Aortic regurgitation index defines severity of peri-prosthetic regurgitation and predicts outcome in patients after transcatheter aortic valve implantation. J Am Coll Cardiol. 2012;59: 1134–41. 10.1016/j.jacc.2011.11.048 22440213

[21] Vasa-Nicotera M, Sinning JM, Chin D, Lim TK, Spyt T, Jilaihawi H, et al Impact of paravalvular leakage on outcome in patients after transcatheter aortic valve implantation. JACC Cardiovasc Interv. 2012;5: 858–65. 10.1016/j.jcin.2012.04.011 22917458

[22] Wendler O, Walther T, Schroefel H, Lange R, Treede H, Fusari M, et al Transapical aortic valve implantation: mid-term outcome from the SOURCE registry. Eur J Cardiothorac Surg. 2013;43: 505–11; discussion 11–2. 10.1093/ejcts/ezs297 22648920

[23] Chopard R, Meneveau N, Chocron S, Gilard M, Laskar M, Eltchaninoff H, et al Impact of chronic obstructive pulmonary disease on Valve Academic Research Consortium-defined outcomes after transcatheter aortic valve implantation (from the FRANCE 2 Registry). Am J Cardiol. 2014;113: 1543–9. 10.1016/j.amjcard.2014.01.432 24630784

[24] Munoz-Garcia AJ, del Valle R, Trillo-Nouche R, Elizaga J, Gimeno F, Hernandez-Antolin R, et al The Ibero-American transcatheter aortic valve implantation registry with the CoreValve prosthesis. Early and long-term results. Int J Cardiol. 2013;169: 359–65. 10.1016/j.ijcard.2013.09.006 24128731

[25] Godino C, Maisano F, Montorfano M, Latib A, Chieffo A, Michev I, et al Outcomes after transcatheter aortic valve implantation with both Edwards-SAPIEN and CoreValve devices in a single center: the Milan experience. JACC Cardiovasc Interv. 2010;3: 1110–21. 10.1016/j.jcin.2010.09.012 21087745

[26] Hayashida K, Lefevre T, Chevalier B, Hovasse T, Romano M, Garot P, et al Impact of post-procedural aortic regurgitation on mortality after transcatheter aortic valve implantation. JACC Cardiovasc Interv. 2012;5: 1247–56. 10.1016/j.jcin.2012.09.003 23257373

[27] Sinning JM, Scheer AC, Adenauer V, Ghanem A, Hammerstingl C, Schueler R, et al Systemic inflammatory response syndrome predicts increased mortality in patients after transcatheter aortic valve implantation. Eur Heart J. 2012;33: 1459–68. 10.1093/eurheartj/ehs002 22285582

[28] Kamga M, Boland B, Cornette P, Beeckmans M, De Meester C, Chenu P, et al Impact of frailty scores on outcome of octogenarian patients undergoing transcatheter aortic valve implantation. Acta Cardiol. 2013;68: 599–606. 24579438 10.1080/ac.68.6.8000007

[29] Dumonteil N, van der Boon RM, Tchetche D, Chieffo A, Van Mieghem NM, Marcheix B, et al Impact of preoperative chronic kidney disease on short- and long-term outcomes after transcatheter aortic valve implantation: a Pooled-RotterdAm-Milano-Toulouse In Collaboration Plus (PRAGMATIC-Plus) initiative substudy. Am Heart J. 2013;165: 752–60. 10.1016/j.ahj.2012.12.013 23622912

[30] Zahn R, Gerckens U, Linke A, Sievert H, Kahlert P, Hambrecht R, et al Predictors of one-year mortality after transcatheter aortic valve implantation for severe symptomatic aortic stenosis. Am J Cardiol. 2013;112: 272–9. 10.1016/j.amjcard.2013.03.024 23578349

[31] Urena M, Webb JG, Tamburino C, Munoz-Garcia AJ, Cheema A, Dager AE, et al Permanent pacemaker implantation after transcatheter aortic valve implantation: impact on late clinical outcomes and left ventricular function. Circulation. 2014;129: 1233–43. 10.1161/CIRCULATIONAHA.113.005479 24370552

[32] Panico C, Pagnotta P, Mennuni M, Corrada E, Barbaro C, Rossi M, et al Predictors of mortality in patients undergoing percutaneous aortic valve implantation. Minerva Cardioangiol. 2012;60: 561–71. 23147434

[33] Katsanos S, Yiu KH, Clavel MA, Rodes-Cabau J, Leong D, van der Kley F, et al Impact of valvuloarterial impedance on 2-year outcome of patients undergoing transcatheter aortic valve implantation. J Am Soc Echocardiogr. 2013;26: 691–8. 10.1016/j.echo.2013.04.003 23669595

[34] Tamburino C, Capodanno D, Ramondo A, Petronio AS, Ettori F, Santoro G, et al Incidence and predictors of early and late mortality after transcatheter aortic valve implantation in 663 patients with severe aortic stenosis. Circulation. 2011;123: 299–308. 10.1161/CIRCULATIONAHA.110.946533 21220731

[35] Barbanti M, Binder RK, Dvir D, Tan J, Freeman M, Thompson CR, et al Prevalence and impact of preoperative moderate/severe tricuspid regurgitation on patients undergoing transcatheter aortic valve replacement. Catheter Cardiovasc Interv. 2014; 10.1002/ccd.25512 24740834

[36] Moat NE, Ludman P, de Belder MA, Bridgewater B, Cunningham AD, Young CP, et al Long-term outcomes after transcatheter aortic valve implantation in high-risk patients with severe aortic stenosis: the U.K. TAVI (United Kingdom Transcatheter Aortic Valve Implantation) Registry. J Am Coll Cardiol. 2011;58: 2130–8. 10.1016/j.jacc.2011.08.050 22019110

[37] Seiffert M, Schnabel R, Conradi L, Diemert P, Schirmer J, Koschyk D, et al Predictors and outcomes after transcatheter aortic valve implantation using different approaches according to the valve academic research consortium definitions. Catheter Cardiovasc Interv. 2013;82: 640–52. 10.1002/ccd.24751 23172652

[38] Lucon A, Oger E, Bedossa M, Boulmier D, Verhoye JP, Eltchaninoff H, et al Prognostic implications of pulmonary hypertension in patients with severe aortic stenosis undergoing transcatheter aortic valve implantation: study from the FRANCE 2 registry. Circ Cardiovasc Interv. 2014;7: 240–7. 10.1161/CIRCINTERVENTIONS.113.000482 24569597

[39] Heinz A, DeCillia M, Feuchtner G, Mueller S, Bartel T, Friedrich G, et al Relative amplitude index: a new tool for hemodynamic evaluation of periprosthetic regurgitation after transcatheter valve implantation. J Thorac Cardiovasc Surg. 2014;147: 1021–8, 9.e1–2. 10.1016/j.jtcvs.2013.11.011 24342900

[40] Webb JG, Altwegg L, Boone RH, Cheung A, Ye J, Lichtenstein S, et al Transcatheter aortic valve implantation: impact on clinical and valve-related outcomes. Circulation. 2009;119: 3009–16. 10.1161/CIRCULATIONAHA.108.837807 19487594

[41] Nuis RJ, Dager AE, van der Boon RM, Jaimes MC, Caicedo B, Fonseca J, et al Patients with aortic stenosis referred for TAVI: treatment decision, in-hospital outcome and determinants of survival. Neth Heart J. 2012;20: 16–23. 10.1007/s12471-011-0224-z 22167520 PMC3247629

[42] Drews T, Pasic M, Buz S, d'Ancona G, Dreysse S, Kukucka M, et al Transcatheter aortic valve implantation in very high-risk patients with EuroSCORE of more than 40%. Ann Thorac Surg. 2013;95: 85–93. 10.1016/j.athoracsur.2012.08.055 23141527

[43] Yamamoto M, Mouillet G, Meguro K, Gilard M, Laskar M, Eltchaninoff H, et al Clinical results of transcatheter aortic valve implantation in octogenarians and nonagenarians: insights from the FRANCE-2 registry. Ann Thorac Surg. 2014;97: 29–36. 10.1016/j.athoracsur.2013.07.100 24140210

[44] Saia F, Ciuca C, Taglieri N, Marrozzini C, Savini C, Bordoni B, et al Acute kidney injury following transcatheter aortic valve implantation: incidence, predictors and clinical outcome. Int J Cardiol. 2013;168: 1034–40. 10.1016/j.ijcard.2012.10.029 23164594

[45] Conrotto F, D'Ascenzo F, Giordana F, Salizzoni S, Tamburino C, Tarantini G, et al Impact of diabetes mellitus on early and midterm outcomes after transcatheter aortic valve implantation (from a multicenter registry). Am J Cardiol. 2014;113: 529–34. 10.1016/j.amjcard.2013.10.025 24315111

[46] Tamburino C, Barbanti M, Capodanno D, Mignosa C, Gentile M, Aruta P, et al Comparison of complications and outcomes to one year of transcatheter aortic valve implantation versus surgical aortic valve replacement in patients with severe aortic stenosis. Am J Cardiol. 2012;109: 1487–93. 10.1016/j.amjcard.2012.01.364 22356793

[47] Van Belle E, Juthier F, Susen S, Vincentelli A, Iung B, Dallongeville J, et al Postprocedural aortic regurgitation in balloon-expandable and self-expandable transcatheter aortic valve replacement procedures: analysis of predictors and impact on long-term mortality: insights from the FRANCE2 Registry. Circulation. 2014;129: 1415–27. 10.1161/CIRCULATIONAHA.113.002677 24566199

[48] Nguyen TC, Babaliaros VC, Razavi SA, Kilgo PD, Guyton RA, Devireddy CM, et al Impact of varying degrees of renal dysfunction on transcatheter and surgical aortic valve replacement. J Thorac Cardiovasc Surg. 2013;146: 1399–406; discussion 13406–7. 10.1016/j.jtcvs.2013.07.065 24075566

[49] Yamamoto M, Hayashida K, Mouillet G, Hovasse T, Chevalier B, Oguri A, et al Prognostic value of chronic kidney disease after transcatheter aortic valve implantation. J Am Coll Cardiol. 2013;62: 869–77. 10.1016/j.jacc.2013.04.057 23707321

[50] Lange R, Bleiziffer S, Mazzitelli D, Elhmidi Y, Opitz A, Krane M, et al Improvements in transcatheter aortic valve implantation outcomes in lower surgical risk patients: a glimpse into the future. J Am Coll Cardiol. 2012;59: 280–7. 10.1016/j.jacc.2011.10.868 22196885

[51] Houthuizen P, Van Garsse LA, Poels TT, de Jaegere P, van der Boon RM, Swinkels BM, et al Left bundle-branch block induced by transcatheter aortic valve implantation increases risk of death. Circulation. 2012;126: 720–8. 10.1161/CIRCULATIONAHA.112.101055 22791865

[52] Gotzmann M, Korten M, Bojara W, Lindstaedt M, Rahlmann P, Mugge A, et al Long-term outcome of patients with moderate and severe prosthetic aortic valve regurgitation after transcatheter aortic valve implantation. Am J Cardiol. 2012;110: 1500–6. 10.1016/j.amjcard.2012.07.010 22863177

[53] Codner P, Assali A, Dvir D, Vaknin-Assa H, Porat E, Shapira Y, et al Two-year outcomes for patients with severe symptomatic aortic stenosis treated with transcatheter aortic valve implantation. Am J Cardiol. 2013;111: 1330–6. 10.1016/j.amjcard.2013.01.275 23415022

[54] Sabate M, Canovas S, Garcia E, Hernandez Antolin R, Maroto L, Hernandez JM, et al In-hospital and mid-term predictors of mortality after transcatheter aortic valve implantation: data from the TAVI National Registry 2010–2011. Rev Esp Cardiol (Engl Ed). 2013;66: 949–58. 10.1016/j.rec.2013.07.003 24774108

[55] Linke A, Wenaweser P, Gerckens U, Tamburino C, Bosmans J, Bleiziffer S, et al Treatment of aortic stenosis with a self-expanding transcatheter valve: the International Multi-centre ADVANCE Study. Eur Heart J. 2014; 10.1093/eurheartj/ehu162 24682842

[56] Unbehaun A, Pasic M, Drews T, Penkalla A, Dreysse S, Klein C, et al Transapical aortic valve implantation: predictors of survival up to 5 years in 730 patients. An update. Eur J Cardiothorac Surg. 2014; 10.1093/ejcts/ezu069 24599161

[57] Le Ven F, Freeman M, Webb J, Clavel MA, Wheeler M, Dumont E, et al Impact of low flow on the outcome of high-risk patients undergoing transcatheter aortic valve replacement. J Am Coll Cardiol. 2013;62: 782–8. 10.1016/j.jacc.2013.05.044 23770162

[58] Rodes-Cabau J, Webb JG, Cheung A, Ye J, Dumont E, Osten M, et al Long-term outcomes after transcatheter aortic valve implantation: insights on prognostic factors and valve durability from the Canadian multicenter experience. J Am Coll Cardiol. 2012;60: 1864–75. 10.1016/j.jacc.2012.08.960 23062535

[59] Moretti C, D'Amico M, D'Ascenzo F, Colaci C, Salizzoni S, Tamburino C, et al Impact on prognosis of periprocedural bleeding after TAVI: mid-term follow-up of a multicenter prospective study. J Interv Cardiol. 2014;27: 293–9. 10.1111/joic.12115 24701998

[60] Rau S, Wessely M, Lange P, Kupatt C, Steinbeck G, Fischereder M, et al Transcatheter aortic valve implantation in dialysis patients. Nephron Clin Pract. 2012;120: c86–90. 10.1159/000335781 22377618

[61] London GM, Pannier B, Marchais SJ, Guerin AP. Calcification of the aortic valve in the dialyzed patient. J Am Soc Nephrol. 2000;11: 778–83. 10752538 10.1681/ASN.V114778

[62] Aregger F, Wenaweser P, Hellige GJ, Kadner A, Carrel T, Windecker S, et al Risk of acute kidney injury in patients with severe aortic valve stenosis undergoing transcatheter valve replacement. Nephrol Dial Transplant. 2009;24: 2175–9. 10.1093/ndt/gfp036 19211648

[63] Soslau G, Brodsky I, Putatunda B, Parker J, Schwartz AB. Selective reduction of serotonin storage and ATP release in chronic renal failure patients platelets. Am J Hematol. 1990;35: 171–8. 2220759 10.1002/ajh.2830350306

[64] Gummert JF, Bucerius J, Walther T, Doll N, Falk V, Schmitt DV, et al Requirement for renal replacement therapy in patients undergoing cardiac surgery. Thorac Cardiovasc Surg. 2004;52: 70–6. 15103578 10.1055/s-2004-817806

[65] McCullough PA. Contrast-induced acute kidney injury. J Am Coll Cardiol. 2008;51: 1419–28. 10.1016/j.jacc.2007.12.035 18402894

[66] Cronin RE. Contrast-induced nephropathy: pathogenesis and prevention. Pediatr Nephrol. 2010;25: 191–204. 10.1007/s00467-009-1204-z 19444480

[67] Van Linden A, Kempfert J, Rastan AJ, Holzhey D, Blumenstein J, Schuler G, et al Risk of acute kidney injury after minimally invasive transapical aortic valve implantation in 270 patients. Eur J Cardiothorac Surg. 2011;39: 835–42; discussion 42–3. 10.1016/j.ejcts.2010.11.034 21186126

[68] Yamamoto M, Hayashida K, Mouillet G, Chevalier B, Meguro K, Watanabe Y, et al Renal function-based contrast dosing predicts acute kidney injury following transcatheter aortic valve implantation. JACC Cardiovasc Interv. 2013;6: 479–86. 10.1016/j.jcin.2013.02.007 23702012

[69] Madershahian N, Scherner M, Liakopoulos O, Rahmanian P, Kuhn E, Hellmich M, et al Renal impairment and transapical aortic valve implantation: impact of contrast medium dose on kidney function and survival. Eur J Cardiothorac Surg. 2012;41: 1225–32. 10.1093/ejcts/ezr199 22219473

