# Supplementary figures and images for: Impact of Renal Dysfunction on Mid-Term Outcome after Transcatheter Aortic Valve Implantation: A Systematic Review and Meta-Analysis
Source: PLoS One. 2015 Mar 20;10(3):e0119817. doi: 10.1371/journal.pone.0119817 (PMC4368625; doi:10.1371/journal.pone.0119817)

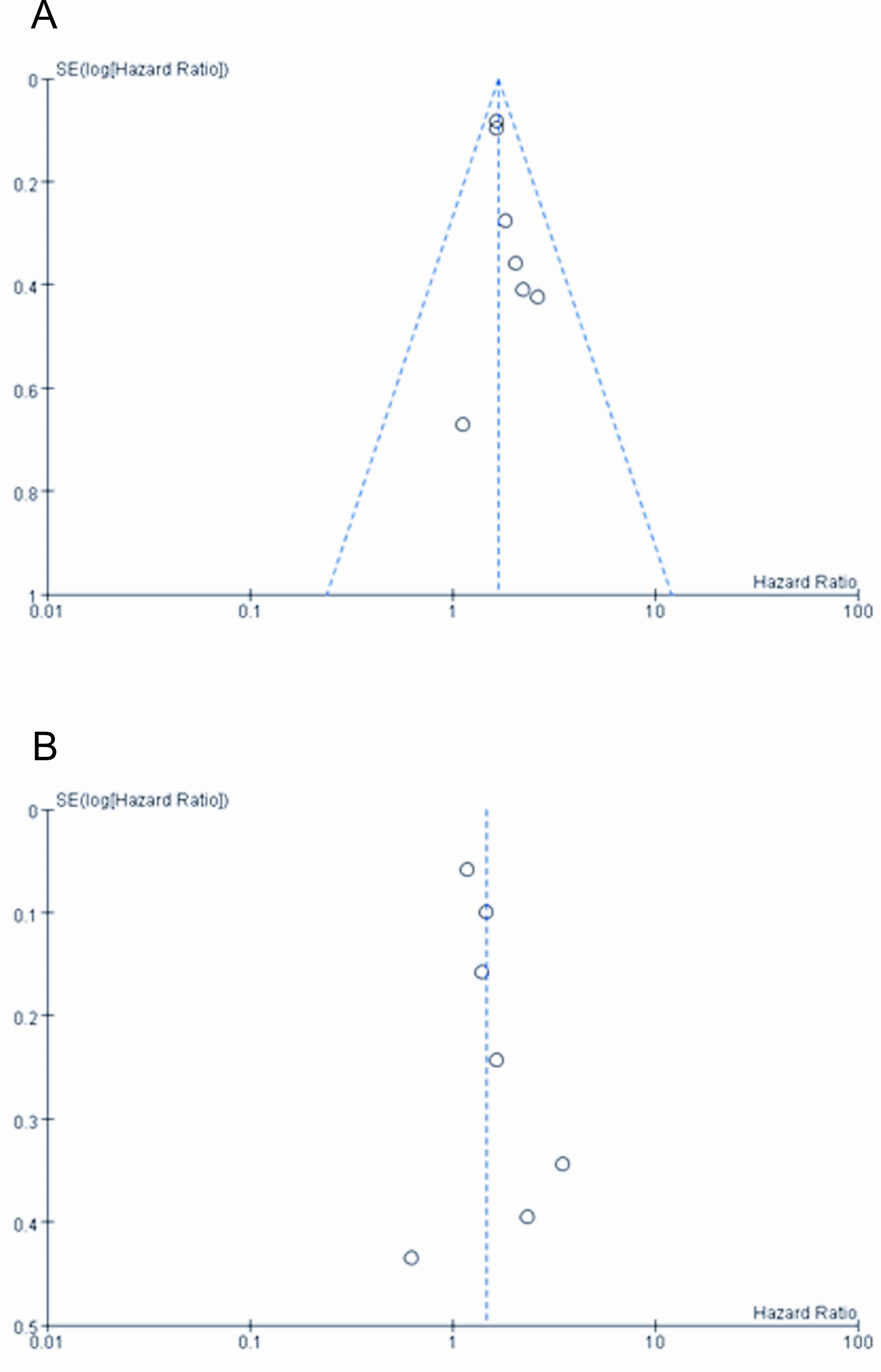

Supplement: S1 Fig — A, Comparison in univariable model (Begg’s test: P = 0.23; Egger’s test: P = 0.208; Trim and Fill Analysis not performed). B, Comparison in multivariable model. (Begg’s test: P = 0.548; Egger’s test: P = 0.215; Trim and Fill Analysis not performed). (TIF) [file pone.0119817.s002.tif]

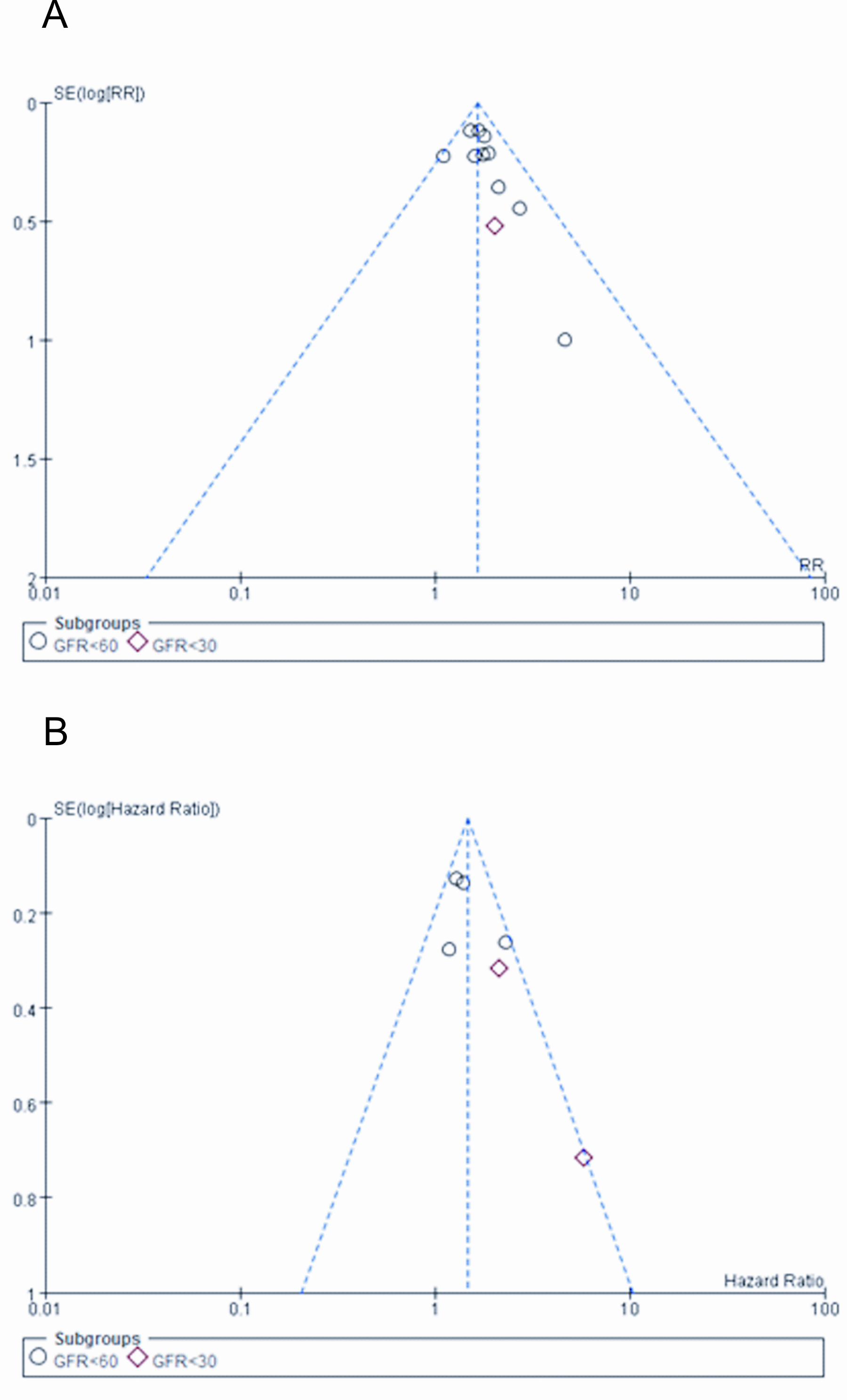

Supplement: S2 Fig — A, Comparison in univariable model. (Begg’s test: P = 0.119; Egger’s test: P = 0.129; Trim and Fill Analysis not performed). B, Comparison in multivariable model. (Begg’s test: P = 0.133; Egger’s test: P = 0.06; Trim and Fill Analysis: Pooled estimate = 0.306, P<0.001). (TIF) [file pone.0119817.s003.tif]

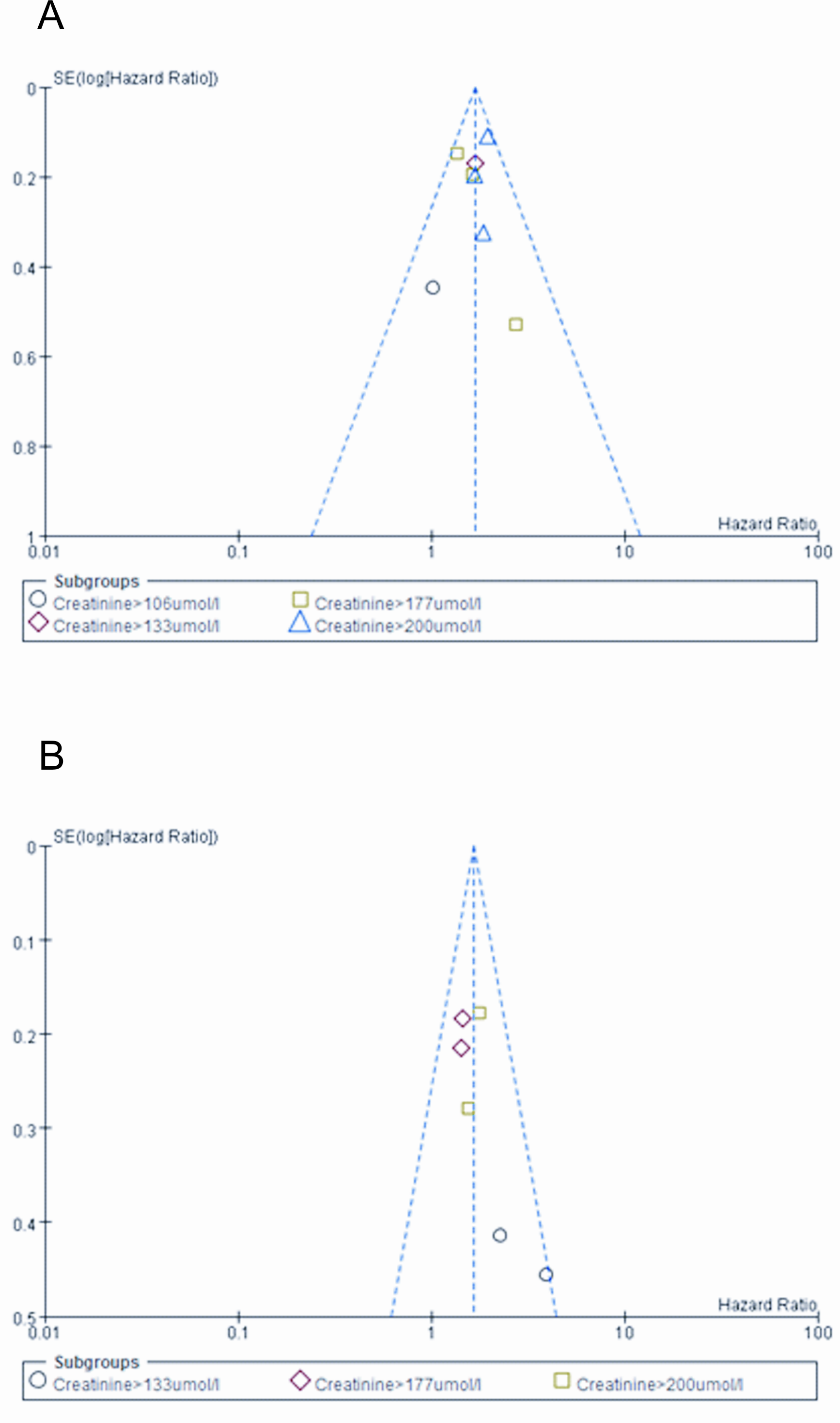

Supplement: S3 Fig — A, Comparison in univariable model. (Begg’s test: P = 0.711; Egger’s test: P = 0.711; Trim and Fill Analysis not performed). B, Comparison in multivariable model. (Begg’s test: P = 0.133; Egger’s test: P = 0.086; Trim and Fill Analysis: Pooled estimate = 0.436, P<0.001). (TIF) [file pone.0119817.s004.tif]

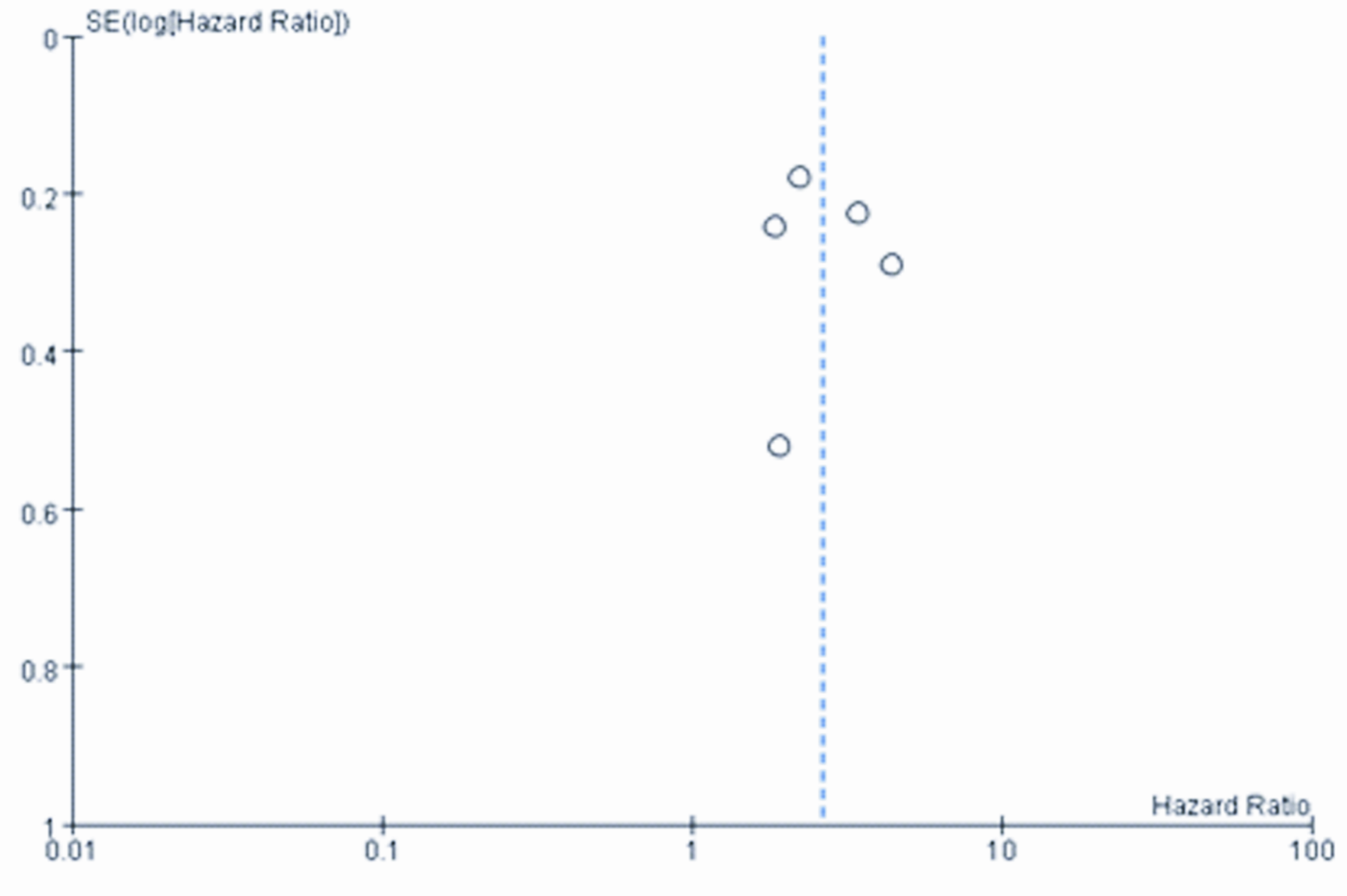

Supplement: S4 Fig — Begg’s test: P = 0.806; Egger’s test: P = 0.841; Trim and Fill Analysis not performed. (TIF) [file pone.0119817.s005.tif]

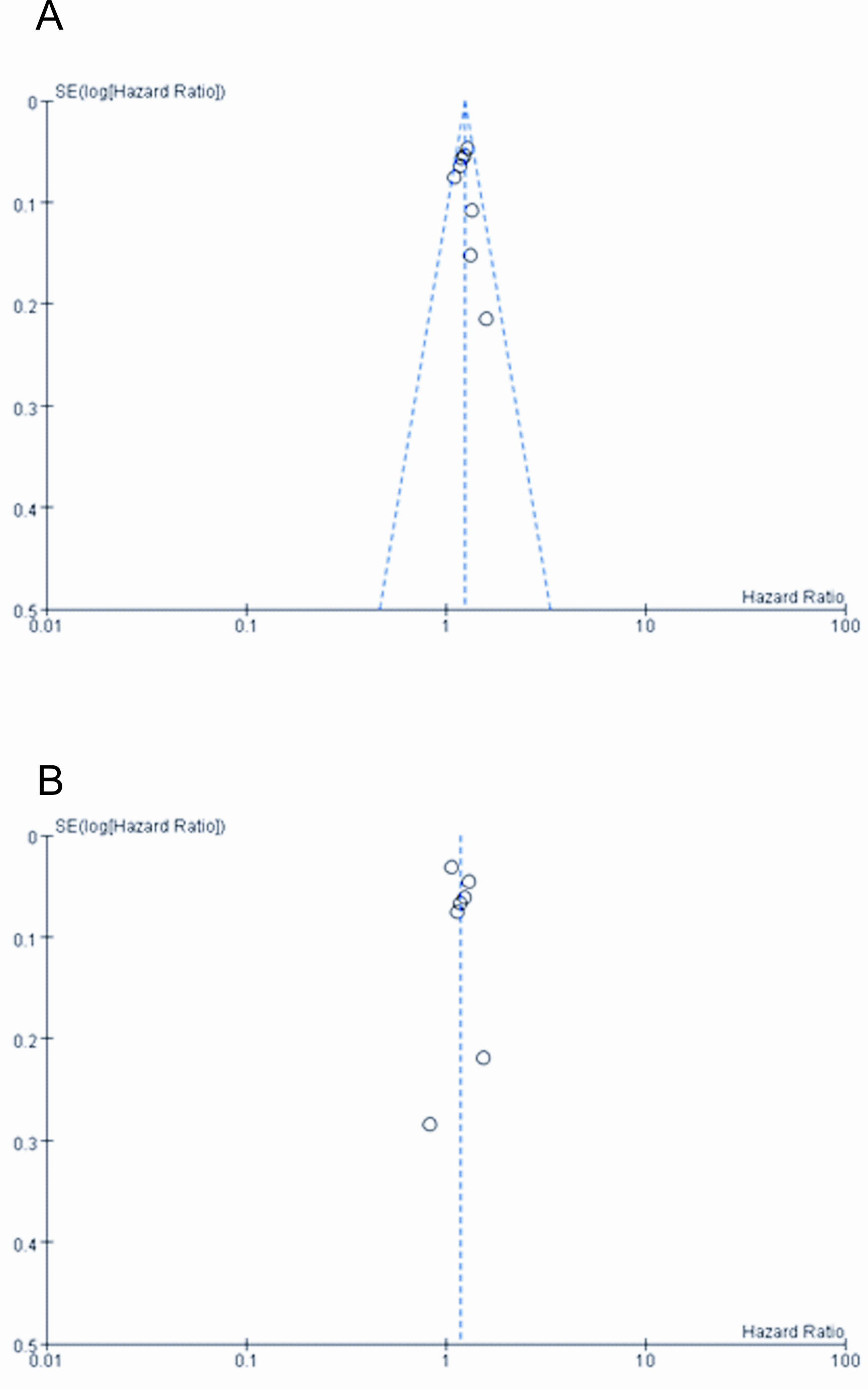

Supplement: S5 Fig — A, Preformed in univariable model. (Begg’s test: P = 0.902; Egger’s test: P = 0.430; Trim and Fill Analysis not performed). B, Preformed in multivariable model. (Begg’s test: P = 0.764; Egger’s test: P = 0.507; Trim and Fill Analysis not performed). (TIF) [file pone.0119817.s006.tif]
